# CD138 expression in the endometrium associates with endometrial timing and inflammatory status but not microbiota composition

**DOI:** 10.1093/humrep/deag032

**Published:** 2026-03-20

**Authors:** J Odendaal, K Fishwick, G D S Correia, Y S Lee, K Makwana, N Black, J Southcombe, J Thornton, K Larsen, Q Hussain, A Hawkes, A Kandiyil, J Muter, P J Brighton, P Vrljicak, E Lucas, I Granne, G Bouliotis, P R Bennett, J Brosens, D A MacIntyre, S Quenby

**Affiliations:** Clinical Sciences Research Laboratories, Division of Biomedical Sciences, Tommy’s National Centre for Miscarriage Research, Warwick Medical School, University of Warwick, Coventry, UK; University Hospitals Coventry & Warwickshire, Coventry, UK; Clinical Sciences Research Laboratories, Division of Biomedical Sciences, Tommy’s National Centre for Miscarriage Research, Warwick Medical School, University of Warwick, Coventry, UK; March of Dimes Prematurity Research Centre, Imperial College London, London, UK; Institute for Reproductive and Developmental Biology, Imperial College London, London, UK; March of Dimes Prematurity Research Centre, Imperial College London, London, UK; Institute for Reproductive and Developmental Biology, Imperial College London, London, UK; Clinical Sciences Research Laboratories, Division of Biomedical Sciences, Tommy’s National Centre for Miscarriage Research, Warwick Medical School, University of Warwick, Coventry, UK; Clinical Sciences Research Laboratories, Division of Biomedical Sciences, Tommy’s National Centre for Miscarriage Research, Warwick Medical School, University of Warwick, Coventry, UK; University Hospitals Coventry & Warwickshire, Coventry, UK; Medical Sciences Division, Nuffield Department of Women’s and Reproductive Health, University of Oxford, Oxford, UK; Clinical Sciences Research Laboratories, Division of Biomedical Sciences, Tommy’s National Centre for Miscarriage Research, Warwick Medical School, University of Warwick, Coventry, UK; Clinical Sciences Research Laboratories, Division of Biomedical Sciences, Tommy’s National Centre for Miscarriage Research, Warwick Medical School, University of Warwick, Coventry, UK; Clinical Sciences Research Laboratories, Division of Biomedical Sciences, Tommy’s National Centre for Miscarriage Research, Warwick Medical School, University of Warwick, Coventry, UK; Clinical Sciences Research Laboratories, Division of Biomedical Sciences, Tommy’s National Centre for Miscarriage Research, Warwick Medical School, University of Warwick, Coventry, UK; University Hospitals Coventry & Warwickshire, Coventry, UK; University Hospitals Coventry & Warwickshire, Coventry, UK; Clinical Sciences Research Laboratories, Division of Biomedical Sciences, Tommy’s National Centre for Miscarriage Research, Warwick Medical School, University of Warwick, Coventry, UK; Clinical Sciences Research Laboratories, Division of Biomedical Sciences, Tommy’s National Centre for Miscarriage Research, Warwick Medical School, University of Warwick, Coventry, UK; Clinical Sciences Research Laboratories, Division of Biomedical Sciences, Tommy’s National Centre for Miscarriage Research, Warwick Medical School, University of Warwick, Coventry, UK; School of Medicine and Population Health, University of Sheffield, Sheffield, UK; Medical Sciences Division, Nuffield Department of Women’s and Reproductive Health, University of Oxford, Oxford, UK; Clinical Sciences Research Laboratories, Division of Biomedical Sciences, Tommy’s National Centre for Miscarriage Research, Warwick Medical School, University of Warwick, Coventry, UK; March of Dimes Prematurity Research Centre, Imperial College London, London, UK; Institute for Reproductive and Developmental Biology, Imperial College London, London, UK; Tommy’s National Centre for Miscarriage Research, Imperial College London, London, UK; Clinical Sciences Research Laboratories, Division of Biomedical Sciences, Tommy’s National Centre for Miscarriage Research, Warwick Medical School, University of Warwick, Coventry, UK; University Hospitals Coventry & Warwickshire, Coventry, UK; March of Dimes Prematurity Research Centre, Imperial College London, London, UK; Institute for Reproductive and Developmental Biology, Imperial College London, London, UK; Robinson Research Institute, University of Adelaide, Adelaide, Australia; Clinical Sciences Research Laboratories, Division of Biomedical Sciences, Tommy’s National Centre for Miscarriage Research, Warwick Medical School, University of Warwick, Coventry, UK; University Hospitals Coventry & Warwickshire, Coventry, UK

**Keywords:** recurrent miscarriage, chronic endometritis, endometrium, microbiome, reproductive immunology

## Abstract

**STUDY QUESTION:**

What is the relationship between constitutive CD138 expression in the endometrium and the reproductive tract microbiota composition?

**SUMMARY ANSWER:**

The presence of CD138^+^ cells in endometrial stroma is cycle-dependent and associated with impaired luteal phase endometrial timing but not altered vaginal or endometrial microbial composition.

**WHAT IS KNOWN ALREADY:**

CD138-diagnosed chronic endometritis (CE) is associated with adverse reproductive outcomes including recurrent pregnancy loss (RPL) in uncontrolled studies. However, CD138 is constitutively expressed in the endometrium, potentially confounding the reported associations between CE, adverse endometrial function, and early pregnancy loss.

**STUDY DESIGN, SIZE, DURATION:**

Translational cohort study of a subset of 103 samples derived from 737 women embedded within the CERM trial, a double-blinded, randomized interventional trial evaluating the impact of pre-pregnancy antibiotic treatment for CE in RPL patients.

**PARTICIPANTS/MATERIALS, SETTING, METHODS:**

Women aged ≥18 to <42 years, with a history of two or more first-trimester consecutive miscarriages were recruited from specialist RPL clinics. Endometrial biopsies, vaginal, ectocervical, and endometrial swabs were obtained 10 ± 4 days following a positive home ovulation test. Additional samples, including proliferative endometrium, were obtained from the Tommy’s National Reproductive Health Biobank. Endometrial biopsies were processed for CD138 expression analysis and immunohistochemistry (IHC), histological dating based on Noyes’ criteria, and molecular timing analysis. Metataxonomic profiling of microbiota was performed by sequencing of bacterial 16S ribosomal RNA genes alongside cytokine analysis.

**MAIN RESULTS AND THE ROLE OF CHANCE:**

IHC revealed three patterns of CD138 immunoreactivity: predominantly membranous punctate staining, predominantly diffuse staining, and a mixed pattern. CD138 is constitutively expressed on the basolateral membrane of glandular epithelial cells and a subset of non-immune stromal cells. Stromal expression was very high (>200 CD138-positive stromal cells/10 mm^2^) in 26 out of 27 proliferative endometrial samples. While CD138 immunoreactivity in the stroma declines markedly following ovulation (Mann–Whitney *U*-test; *P* < 0.005), gene expression analysis demonstrated a reduction in *SDC1* expression encoding CD138/syndecan-1, across the menstrual cycle. When compared to CD138-negative samples, conspicuous diffuse staining in the stromal compartment was associated with significantly earlier endometrial histological dating (*P* < 0.01) and lower molecular timing ratios (*P* < 0.01). Poor correlation between CD138 and immunoreactivity was demonstrated. Sequencing of paired vaginal and ectocervical swabs and endometrial Tao brush samples collected from 114 patients demonstrated tightly interconnected microbial composition throughout the reproductive tract. No significant difference in vaginal, ectocervical, or endometrial community state type with CD138 expression was demonstrated. Analysis of supernatants of vaginal and ectocervical swabs and Tao Brush revealed an inverse correlation between the severity of stromal CD138 immunoreactivity in endometrial stroma and secreted levels of IL-10, TNF-α, and VEGF (q < 0.05).

**LARGE SCALE DATA:**

Microbial and Metataxonomic raw data are available in the European Nucleotide Archive (Projects PRJEB83331 and PRJEB83332).

**LIMITATIONS, REASONS FOR CAUTION:**

This study relied on patient-reported ovulation-based timing. This was, however, associated with the provision of validated ovulation tests. In addition, the study is limited by lack of collection of data on the underlying fertility-related co-morbidities due to exclusion of known contributory co-morbidities at the point of recruitment.

**WIDER IMPLICATIONS OF THE FINDINGS:**

This study challenges the purported relationship between CD138^+^ CE and the pathophysiology of CE-associated RPL. The findings indicate endometrial CD138 levels are non-immune and non-bacterial driven and are associated with endometrial immaturity. CD138-based CE testing and treatment should not be performed outside of a research context.

**STUDY FUNDING/COMPETING INTEREST(S):**

Funding was provided by the Efficacy and Mechanism Evaluation (EME) Programme a National Institute for Health and Care Research and Medical Research Council partnership (17/60/22). Further funding was from Tommy’s National Centre for Miscarriage Research, and the Imperial National Institute for Health and Care Research Biomedical Research Centre Pregnancy and Prematurity Theme. G.D.S.C. is supported by the Genesis Research Trust. All authors report no direct conflict of interest.

**TRIAL REGISTRATION NUMBER:**

ISRCTN23947730.

## Introduction

Early pregnancy loss affects up to 15% of all pregnancies (Quenby *et al.*, 2021). Recurrent pregnancy loss (RPL) defined as the loss of two or more pregnancies affects up to 5% of couples (Bender Atik *et al.*, 2018). The aetiology of the disorder remains poorly understood and is likely multifactorial. Chronic endometritis (CE) is characterized by asymptomatic low-grade inflammation of the endometrium and is associated with RPL (Groth, 2018). While the underlying causal factors of CE remain poorly understood, some studies have reported an association between CE, microbial dysbiosis, and pathogen colonization of the endometrium (Cicinelli *et al.*, 2014; Kitaya *et al.*, 2017) but these relationships are inconsistently observed, with a histological concordance of 46% seen in one frequently cited study (Moreno *et al.*, 2018).

CE is characterized by the infiltration of the endometrial stroma with plasma cells (Groth, 2018). Traditionally, plasma cell infiltration has been assessed using haematoxylin and eosin (H&E) based diagnostic approaches but contemporary diagnosis relies on the detection of the purported plasma cell marker, CD138, which has better inter-/intra-observer variability (Liu *et al.*, 2018). CD138-based diagnosis of CE has also been associated with RPL (Mitter *et al.*, 2021) with non-randomized, non-placebo-controlled studies reporting improved reproductive outcomes in women treated with antibiotics, further implicating an infectious aetiology in the pathogenesis of CE (Cicinelli *et al.*, 2014; McQueen *et al.*, 2015; Mitter *et al.*, 2021).

However, there exists major heterogeneity in diagnostic thresholds for CE based on CD138 immunohistochemistry (IHC) (Huang *et al.*, 2020). These discrepancies have resulted in marked differences in the reported prevalence of CE with rates ranging from 9% to 66% (McQueen *et al.*, 2015), which can be partly attributed to the use of narrow or broad diagnostic criterion (Huang *et al.*, 2020). Diagnostic differences are also reflected in reproductive prognostic outcomes with the odds ratio of both clinical pregnancy rate (OR 1.83; 95% CI: 1.18–2.8) and live birth rate (OR 2.08; 95% CI: 1.43–3.02) affected by the diagnostic criterion used (Huang *et al.*, 2020).

CD138 (syndecan-1; *SDC1*) is a member of the transmembrane heparan sulphate proteoglycan family and is constitutively expressed in human endometrium (Germeyer *et al.*, 2007). However, like other syndecans, its expression varies across the menstrual cycle. While whole tissue expression of *SDC1* increases as the endometrium progresses from the proliferative to secretory phase of the menstrual cycle (Germeyer *et al.*, 2007), stromal CD138 immunoreactivity decreases during this period (Lai *et al.*, 2007). Despite being widely used as a marker of plasma cell invasion in the endometrial stroma, the biological relevance of CD138 in the pathogenesis of CE remains unclear. This is exemplified by recent findings indicating no relationship between CD138 levels and live birth prediction (Herlihy *et al.*, 2022). Nevertheless, it is widely accepted that endometrial derangement interfering with the formation of the window of receptivity for embryo implantation is a cause of RPL (Ewington *et al.*, 2019). Therefore, in this exploratory study, we aimed to evaluate the relationship between constitutive CD138 expression in the endometrium and endometrial characterization including the reproductive tract microbiota.

## Materials and methods

### Participant recruitment and sample processing

Ethical approval for this study was obtained from the NHS Research Ethics Committee (REC) Northwest-Haydock (19/NW/0462). Participants were consented and recruited from the Chronic Endometritis and Recurrent Miscarriage (CERM) trial, the protocol of which has previously been published (Odendaal *et al.*, 2023). In brief, women aged ≥18 to <42 years, with a history of two or more consecutive first-trimester miscarriages, were recruited to the trial from a specialist RPL clinic. Women were excluded if they experienced a live birth, termination or stillbirth between miscarriages, had a history of a known cause of RPL or systemic inflammatory conditions. Women would not previously have undergone uterine cavity evaluation or CE testing as part of standard National Health Service care. Where this was previously undertaken and a known cause of RPL was identified, they were excluded. Participants were given; access to weekday telephone advice, menstrual diaries, ovulation kits to detect their urinary LH surge, condoms, and advised to avoid conceiving. Women underwent a timed endometrial biopsy 10 ± 4 days following ovulation. In the event that ovulation testing was inconclusive, screening was performed on Day 23 ± 3 of the menstrual cycle. All participants required a negative pregnancy test at the point of sample collection. A high vaginal and endocervical microbial swab were collected. Following this, an endometrial swab was taken using a sheathed Tao Brush (Cook Medical, Bloomington, IN) to minimize cervicovaginal cross-contamination. Swabs were immediately placed on ice and transferred to a −80 °C freezer. An endometrial tissue biopsy was obtained using a Wallach Endocell™ endometrial sampler (Cooper Surgical, Trumbull, CT). Endometrial biopsies were split into either 10% neutral buffered formalin for at least 24 h for histology, or immersed in 2 ml of RNAlater solution (Sigma-Aldrich, St. Louis, MO) and stored at −80°C for analysis of RNA. A subset of samples collected from the first sequential participants recruited was used for experiments described below to avoid bias.

### Study outcomes

This exploratory study had a primary aim of establishing the characteristics of CD138^+^ endometrium. These characteristics were benchmarked against endometrial timing, CD19 immune cell profile, endometrial cytokine profile, and the reproductive tract microbiota.

### Endometrial swab validation

Validation of the method used to obtain an endometrial swab was conducted. This ascertained sampling suitability. Ethical approval for the project was obtained from the Tommy’s National Reproductive Health Biobank (TNRHB) (TSR19-003). The TNRHB has ethical permission for approval of tissue use (NHS REC 18/WA/0356 Wales). Samples were collected from a dedicated implantation research clinic at University Hospitals Coventry and Warwickshire. Samples were collected on Days 6 and 12 post-ovulation in non-hormonally induced menstrual cycles. Each woman underwent multi-site sampling as follows. Using a speculum, a high vaginal swab was taken by gently rotating a VWR liquid Amies swab (VWR™, Lutterworth, UK) within the posterior fornix of the vagina for 30 s. An endocervical swab was then taken by gently rotating a cotton bud within the endocervix for 30 s. A sheathed Tao brush was then inserted into the uterine cavity with the sheath closed on entry through the internal cervical os. The sheath was then retracted, and the brush advanced to the uterine fundus and rotated 360° clockwise followed by a 360° counter-clockwise rotation to effect a sample. The brush was then withdrawn into the sheath and removed to avoid sample contamination before being placed into a sterile microcentrifuge tube and immediately placed on dry ice for transport. For each endometrial Tao brush sample, the tip of the device (ball) and the outer protective sheath (sheath) were carefully separated from the brush and also sequenced. Additional negative controls (2 for each sample type, except for Tao brush ball and sheath where only 1 negative control was prepared) were included to allow for assessment of environmental and kit contamination. An endometrial tissue biopsy was then performed using a Wallach Endocell™ endometrial sampler and immediately placed on dry ice for transport (Cooper Surgical, Trumbull, CT). Samples were transferred to a −80°C freezer for long-term storage.

### Biobank sample acquisition

Additional evaluation of CD138 expression across the menstrual cycle was performed using endometrial samples obtained from the TNRHB. In addition, proliferative phase samples and control tonsillar tissue for IHC assessment were obtained from the Arden tissue bank (Southampton Central REC: 18/SC/0180).

### Mining of data repositories

Data extraction of the Gene Expression Omnibus was performed to derive CD138 (*SDC1*) expression data across cycle. The following datasets were interrogated: whole tissue CD138 expression (Accession number: GDS2052/201287) (Talbi *et al.*, 2006; Barrett *et al.*, 2013) and high-throughput single-cell RNA sequencing (Accession number: GSE111976) (Wang *et al.*, 2020). Testing for normality across the datasets was performed with Shapiro–Wilks normality testing.

### CD138 immunohistochemistry

Formalin-fixed samples were embedded in Surgipath^®^ Formula ‘R’™ paraffin using the Shandon Excelsior ES Tissue processor 96 (ThermoFisher; Waltham, MA, USA). Three micrometre sections were mounted on a glass slide and baked overnight at 60°C. Sections were stained with B-A38 CD138 antibody, purchased prediluted with the fully automated Roche Ventana Discovery XT Autostainer (Roche, F. Hoffmann-La Roche Ltd. Basel, Switzerland). A positive control of tonsillar tissue and a negative control of tonsillar tissue stained by a monoclonal antibody not directed against a known epitope in human tissue were also prepared. Slides were coverslipped and images were digitized on a 3D Histech P150 slide scanner using a 20× objective lens. Staining processes were selected to align with those utilized within clinical pathology.

### CD138 image analysis

Images were reviewed using Case Viewer V2.2 (3DHISTECH Ltd, Budapest, Hungary). Entire sections were reviewed following a previously published methodology (Liu *et al.*, 2018). Sections were reviewed at 20× magnification and positive cells counted with CD138^+^ cell density was calculated per 10 mm^2^. Cases were defined as positive for CE if CD138^+^ cell density was ≥5 CD138^+^ cells/10 mm^2^. Further subcategorization was undertaken based on CD138 cell density as calculated: negative <5 CD138^+^ cells/10 mm^2^, mild 5-20 CD138^+^ cells/10 mm^2^, moderate 21-200 CD138+ cells/10 mm^2^, and severe > 200 CD138^+^ cells/10 mm^2^. Subcategorization in this manner was performed a priori to further understand the impact of differential diagnostic thresholds given previously published work demonstrating differences in CE prevalence and clinical predictiveness dependent on whether a broad or narrow diagnostic criterion was utilized (Huang *et al.*, 2020). In addition, following pilot work in anticipation of the trial, three differential CD138 staining patterns were noted. The first is a uniform peri-membranous punctate expression. The second is a diffuse pan-cellular expression and the third is a mixed pattern encompassing the two described types. To understand the significance of this, staining patterns were visually characterized by the quantifying observer. Where >50% of the staining seen was punctate, this was classified as a punctate pattern and similarly for diffuse. Where equal proportions of pattern were present a mixed pattern was noted. Slides noted as borderline underwent second review by an independent observer.

### CD138 glandular expression quantification

CD138 immunostaining within the endometrial glandular compartment was quantified and localized using H-scoring by two observers (Budwit-Novotny *et al.*, 1986). Images were reviewed using Image J software (Rasband W.S.; National Institute of Health, Maryland, USA). Ten random high-power fields were selected per section. Glands were subsequently reviewed and assigned a score of 0, 1, 2, or 3; where 0 = no staining, 1 = mild staining, 2 = moderate staining, and 3 = strong staining. The total H-score was calculated as the sum of the percentage cells at a given staining intensity multiplied by their strength score (H-score = ΣPi(*i* + 1)) (Budwit-Novotny *et al.*, 1986).

### CD19 staining

Paraffin-embedded samples, generated as above, were sliced into 3 μm sections and mounted on a glass slide. Slides were baked at 60°C overnight. Sections were stained for CD19 with Anti-CD19 Rabbit Monoclonal Antibody (Abcam) in Bond Primary Antibody Diluent (Leica Biosystems) at a concentration of 1:250. Endometrial biopsies were stained on a Leica BOND-MAX Fully Automated IHC Stainer using a Leica Bond Polymer Refine Detection kit. Following deparaffinization, 20 min of heat-induced epitope retrieval with Leica Epitope Retrieval 2 (ER2) was followed by application of primary antibody for 15 min (CD68 for 20 min). A peroxide block was applied for 5 min, and post-primary for 15 min. DAB (3,3′-diaminobenzidine) chromogen was applied for 10 min. Finally, haematoxylin was used as a counterstain and applied for 7 min. Tonsillar tissue was used as a positive control.

### CD19 image analysis

Images were reviewed using Case Viewer V2.2 (3DHISTECH Ltd, Budapest, Hungary). CD19^+^ cells were quantified as described for CD138 image analysis, however, counting was not capped at 200 positive cells. Direct per sample comparison with CD138 expression was performed.

### H&E staining

Paraffin-embedded samples, generated as above, were sliced into 3 μm sections and mounted on a glass slide. Slides were baked at 60°C overnight. Slides then underwent H&E staining using Tissue-Tek^®^ Prisma^®^ Automated Slide Stainer, model 6134 (Sakura Flinetek Inc., CA, USA). Slides were coverslipped in a Tissue-Tek^®^ Prisma^®^ Automated Slide Stainer, model 6134 (Sakura Flinetek Inc. CA, USA). Images were digitized on a 3D Histech P150 slide scanner using a 20× objective lens.

### Histological timing

Digitized H&E images were reviewed using Case Viewer V2.2 (3DHISTECH Ltd, Budapest, Hungary). Entire sections were reviewed and histologically timed according to Noyes endometrial dating criteria (Noyes *et al.*, 1975). A subset of 10% of samples was independently reviewed by a histopathologist to confirm timing category.

### *In situ* hybridization

In situ hybridization was performed using an RNAScope© assay as per the manufacturer’s instructions (Advanced Cell Diagnostics Inc. Newark, USA) (Wang *et al.*, 2012). Formalin-fixed paraffin-embedded 5 μm plated samples were baked at 60°C and deparaffinized using xylene and 100% alcohol. Antigen retrieval with RNAscope^®^ 1X Target Retrieval Reagent at 100°C for 30 min was performed. Samples were treated with RNAscope^®^ Protease Plus and incubated at 40°C for 30 min. Propriety probes for *SDC1* were placed on the sample and incubated at 40° C for 2 h. Amplification was performed as per the manufacturer’s instructions. DAB was applied and samples counterstained with haematoxylin. Slides were immediately transferred into 0.02% ammonia water for bluing and then dried at 60°C for 30 min. Following coverslipping, images were captured via an EVOS FL Auto fluorescence microscope (Life Technologies, Paisley, UK).

### Real-time polymerase chain reaction

Two cubic millimetres of excised RNAlater-preserved tissue were segmented. RNA was isolated from whole endometrial samples. RNA concentration was determined by Nanodrop 2000c spectrophotometer (Thermo Scientific, USA). cDNA synthesis was performed using a QuantiTech Reverse Transcription Kit. RT-qPCR was conducted using an ABI PRISM 7500 Sequence Detection System (Applied Biosystems; Waltham, MA, USA). Primers for *GPX3* and *SCL15A2* were added to the mix. Samples were analysed in triplicate. Non-template controls were run with nuclease-free water replacing cDNA. Ct values were adjusted to a housekeeping gene L19 by the ΔΔCt method. This normalized data were then compared by the sample phenotype of interest. Primer sequences can be seen in Supplementary Table S1.

### Molecular timing analysis

Molecular timing utilizing an adapted two-gene methodology was conducted based upon the ratio of expression of GPX3 and SLC15A2 (Lipecki *et al.*, 2022). These were normalized to a within run sample control and normalized to patient-reported day of cycle.

### Flow-cytometry

Endometrial biopsy digests were thawed in RPMI + 10%FCS media, 2 × 10^6^ cells were resuspended in PBS and stained with 1 µl of 1:1000 dilution Zombie Aqua Viability dye (Biolegend; San Diego, CA) for 10 min in the dark at RT, then incubated with antibodies CD138-AF647, CD45-AF700, CD19-PeCy7, EpCAM-PE (Biolegend; San Diego, CA) for 20 min at 4 °C. Cells were washed in cell staining buffer (Biolegend; San Diego, CA) and acquired immediately on a LSRII Flow cytometry (BD Biosciences; Franklin Lakes, NJ) and data analysed using FACS DIVA (BD Biosciences; Franklin Lakes, NJ, USA) (Supplementary Fig. S1).

### Bacterial DNA extraction

Bacterial DNA extraction for 16S rRNA gene amplification was undertaken utilizing previously published methodology (Macintyre *et al.*, 2015). Laboratory staff were blinded to CD138 status. Cervicovaginal swabs from were thawed on ice and vortexed to ensure resuspension of cervicovaginal fluid. The resulting solution was then transferred to a 2 ml centrifuge tube and centrifuged at 5400×*g* for 10 min at 4 °C. Endometrial Tao Brush samples were immersed in 500 µL filter-sterilized PBS supplemented with 1 mg/ml Protease inhibitor (Sigma Cat 78428) in 2 ml microfuge tube. Endometrial tissue and bacterial cells were loosened and washed off the Tao Brush into the PBS buffer by gentle vortexing and repeated pipetting action. The brush was then removed from the 2 ml microfuge, and the remaining cell suspension in PBS centrifuged at 5400×*g* for 10 min at 4 °C to pellet. The supernatant was stored in −20°C for analysis of cytokines and other soluble immune markers. The subsequent pellet (both swab and Tao Brush) was re-suspended in a lysis buffer comprising 50 µl lysozyme (10 mg/ml), 6 µl mutanolysin (25 000 U/ml), 3 μl lysostaphin (4000 U/ml), 41 μl TE (Tris 10 mM + 50 mM EDTA), 30 μl 12% Triton, and 170 μl of filter sterile PBS, and then incubated for 1 h at 37 °C. Bacterial cells were disrupted using a Tissue Lyser LT (Qiagen) for 1 min at 25 Hz with 0.1 mm diameter acid-washed glass beads. This lysate was purified using QIAmp DNA mini kit (Qiagen, Manchester, UK) and the DNA eluted in 100 μl AE buffer. Amplification of the V1 and V2 hypervariable regions of the 16S rRNA genes (see Supplementary Table S1) was then performed to assess success of the DNA extraction.

### Metataxonomic profiling

Amplicons of the bacterial 16S rRNA gene V1–V2 hypervariable region were obtained using a primer set with four forward primers, 28F-YM (5′-GAGTTTGATCNTGGCTCAG-3′), 28F-Borrellia (5′-GAGTTTGATCCTGGCTTAG-3′), 28F-Chloroflex (5′-GAATTTGATCTTGGTTCAG-3′), and 28F-Bifido (5′-GGGTTCGATTCTGGCTCAG-3′), at a ratio of 4:1:1:1, and a 388R reverse primer (5′-TGCTGCCTCCCGTAGGAGT-3′) (Frank *et al.*, 2008). Sequencing was performed on the Illumina MiSeq platform (Illumina, Inc. San Diego, CA) at Research and Testing Laboratory (Lubbock, TX, USA). Read quality was assessed with FASTQC (*v*0.11.9) and primer sequences removed with cutadapt (*v*4.0). Amplicon sequence variants (ASVs) were identified with the *dada2* denoizing algorithm. A bespoke naïve Bayes classifier was trained using all sequences in the Silva SSU database (release 138) and used for taxonomic classification of the ASVs at species-level (Quast *et al.*, 2013). The denoizing and taxonomic assignment steps were done through a QIIME2 workflow (*v*2022.2.1). Putative contaminant ASVs were identified and removed with the R package decontam (*v*1.14.0), using the prevalence filter (threshold = 0.1) and blank swabs and Tao brushes that underwent the whole DNA extraction workflow using the same reagents as negative controls. This contaminant removal step was not applied to the initial analysis performed with multiple sampling device controls to assess the Tao brush endometrial sampling strategy. ‘Decontaminated’ data were then re-imported into QIIME2, and genera- and species-level counts exported. The VALENCIA classifier was used to classify metataxonomic profiles into community state types (CST). Raw data are available in the European Nucleotide Archive (Projects PRJEB83331 and PRJEB83332). Scripts and coding used for data preprocessing are available in the Github repository supporting this publication (https://github.com/Gscorreia89/cerm-ce).

### Cytokine analysis

Swab and Tao Brush supernatants from the microbial DNA extraction were diluted 1:10 with filter-sterilized PBS supplemented with 1 mg/ml protease inhibitor. Measurements were performed on a Luminex MAGPIX Instrument with the xPONENT 4.3 software (R&D systems). CXCL14/BRAK, IL-10, IL-15, IL-6, SLPI, TNF-α, and VEGF were assayed with a custom 7-plex Human Luminex Discovery Assay kit, and IL-8 with a separate kit from the same supplier. Each sample was measured in duplicate, with standard calibration curves and quality control samples executed as per kit manufacturer instructions.

### Statistical analysis

Statistical analysis was undertaken with Graphpad Prism 9 (Graphpad Software; San Diego, CA, USA). Significance testing was performed utilizing the Mann–Whitney *U*-test for two groups or the Kruskal–Wallis test for >2 with post hoc analysis performed for multi-group significance testing.

Hierarchical clustering analyses were performed in Python (*v3.9.17)* and *scipy (v1.11.2)*, with the *seaborn (v0.12.2)* and *matplotlib (v3.7.2)* packages used to draw heatmap visualizations. Clusters were identified with Ward-linkage and Jensen–Shannon distance. Number of clusters was selected based on maximization of the mean silhouette score. Other statistical analyses of 16S metataxonomic and cytokine profiles were performed in R (*v*4.3.3). Diversity measures were calculated with the *phyloseq* package (*v1.46.0*). Centred-log-ratio transformation was applied with a probabilistic 0 value imputation method, from the *zCompositions* (*v1.5.0.4*) package. Taxa present in less than 5% of the dataset were excluded from subsequent analyses. PERMANOVA estimates were obtained with the *vegan* (*v2.6.6.1*) package *adonis2* function, using the settings method = ‘*euclidean*’, by = ‘*marginal*’, and 1000 permutations. Differential abundance analyses were executed with either ALDEx2 (*v1.34.0*) directly with linear models (when adjusting for variables). Cytokine values were log-transformed after addition of a constant (*c* = 1) offset and modelled as a function of CD138 diagnosis, severity, or expression pattern. The Benjamini–Hochberg false discovery rate correction was used for multiple testing correction in the microbiome and cytokine analyses. Data analysis scripts are available in the supporting GitHub repository (https://github.com/Gscorreia89/cerm-ce).

## Results

### Participant demographics

**Figure 1.**
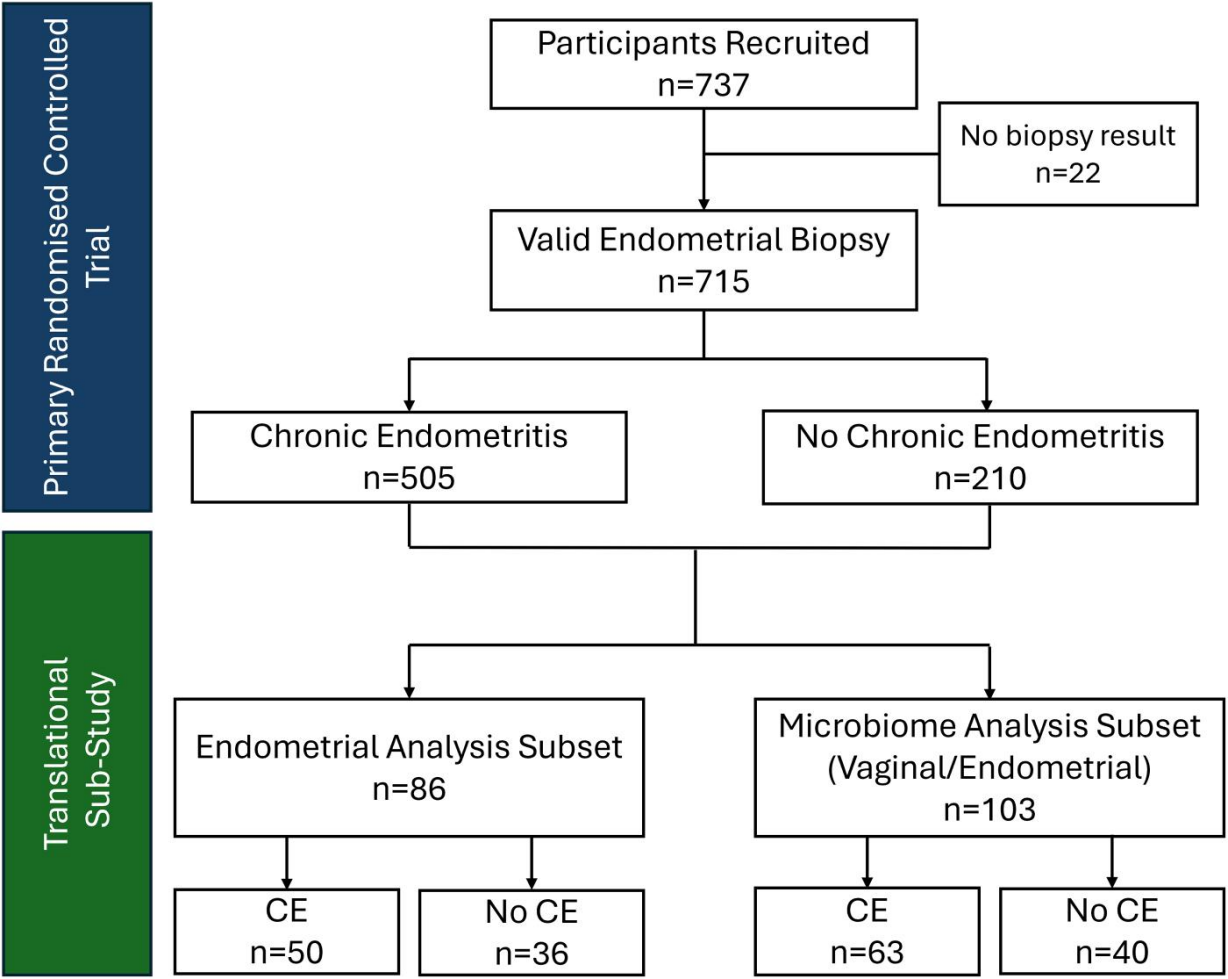
**Participant recruitment and sample use**.

A total of 737 women were recruited into the study. Of these, 715 had biopsy results available with 505 (70.6%) screening positive for CE (CD138^+^ Cell Density >5 CD138^+^ cells/10 mm^2^) and 210 (29.4%) screening negative. Of those positive, 179 (35.4%) demonstrated mild expression, 171 (33.9%) moderate, and 155 (30.7%) severe. A flow diagram summarizing participant recruitment and diagnoses is presented in Fig. 1 and a breakdown of the distribution of CD138 staining is presented in Table 1. Participant grouping was balanced across age, BMI, and number of previous miscarriages. Trial participant demographics are summarized in Table 2. A subset of samples including both CE-positive and CE-negative samples (n = 86) underwent detailed endometrial analysis while 103 samples underwent multi-site microbiome and immune mediator analysis. Sample sizes were selected pragmatically due to the limitations imposed by the SARS-Covid-19 pandemic.

**Table 1.** Participant CD138 expression.

	Punctate	Diffuse	Mixed	Total
Negative	198	6	6	210
Mild	140	19	21	180
Moderate	55	76	40	171
Severe	9	127	18	154
**Total**	402	228	85	715

**Table 2.** Participant demographics.

	Chronic endometritis	No chronic endometritis	*P*-value
Age, mean (95% CI)	34.48 (34.09; 34.86)	34.14 (33.50; 34.75)	0.34
BMI, mean (95% CI)	27.16 (26.63; 27.68)	26.57 (25.84; 27.31)	0.23
Ethnicity, *n* (percentage)			
* White British*	368 (72.9)	159 (75.7)	0.44
* White Irish*	6 (1.2)	3 (1.4)	
* Other White*	57 (11.3)	18 (8.6)	
* White and Black Caribbean*	0 (0.0)	2 (1.0)	
* White and Asian*	1 (0.2)	1 (0.5)	
* Other Mixed*	2 (0.4)	1 (0.5)	
* Indian*	34 (6.7)	15 (7.1)	
* Pakistani*	16 (3.2)	3 (1.4)	
* Bangladeshi*	3 (0.6)	2 (1.0)	
* Other Asian*	7 (1.4)	0 (0.0)	
* Caribbean*	1 (0.2)	2 (1.0)	
* African*	3 (0.6)	1 (0.5)	
* Chinese*	3 (0.6)	2 (1.0)	
* Other ethnic group*	3 (0.6)	1 (0.5)	
* Ethnicity not given*	1 (0.2)	0 (0.0)	
Number of 1st trimester miscarriages, mean (SD)	3.70 (2.11)	3.80 (1.83)	0.51
At least ONE previous livebirth (percentage)	40.6% (205/505)	40.9% (86/210)	0.92
Mean EARLY miscarriage gestation, mean (SD)	6.91 (2.16)	6.69 (2.11)	0.21
Mean LATE miscarriage gestation, mean (SD)	16.71 (3.44)	13.58 (4.52)	<0.01
Mean ANY miscarriage gestation, mean (SD)	7.13 (2.58)	6.79 (2.30)	0.11

### Endometrial stroma CD138 expression is highly variable

**Figure 2.**
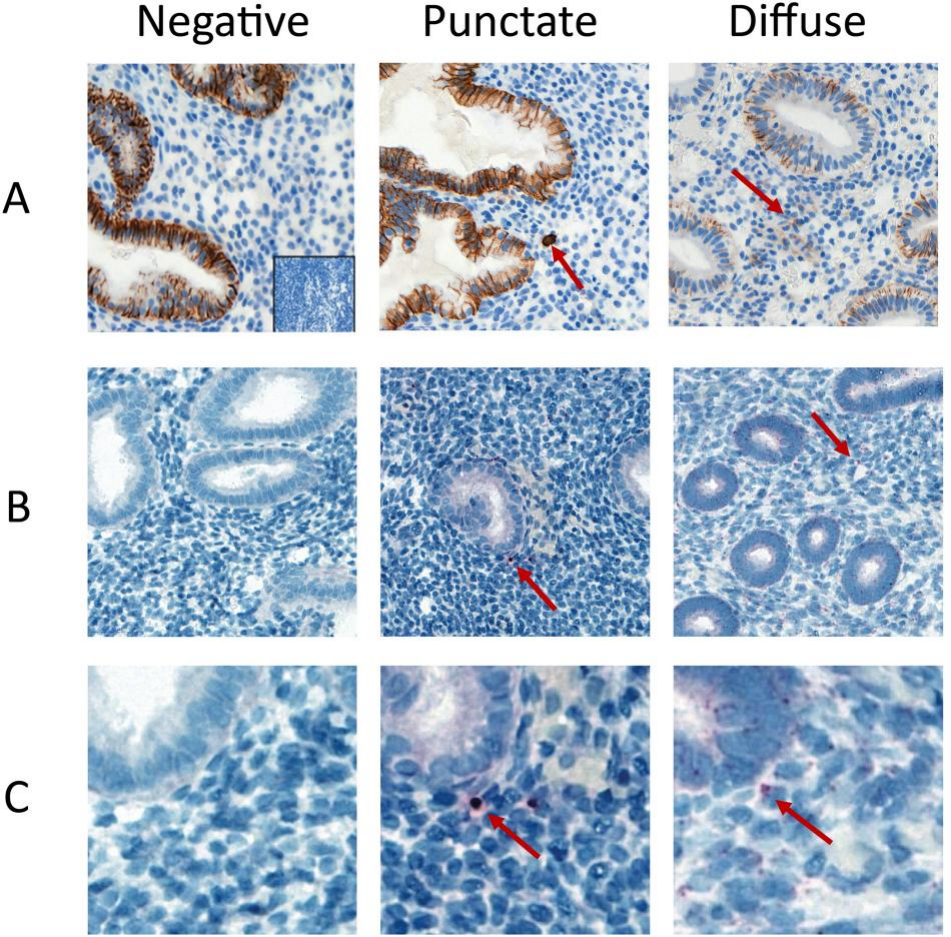
**CD138 staining patterns with small-molecule in situ hybridization validation (smISH).** 38.3× magnification. Row (**A**): CD138 immunohistochemistry. Row (**B**): smISH staining. Row (**C**): Magnification of smISH staining (200%). Arrows indicate exemplar CD138-stained cells.

**Figure 3.**
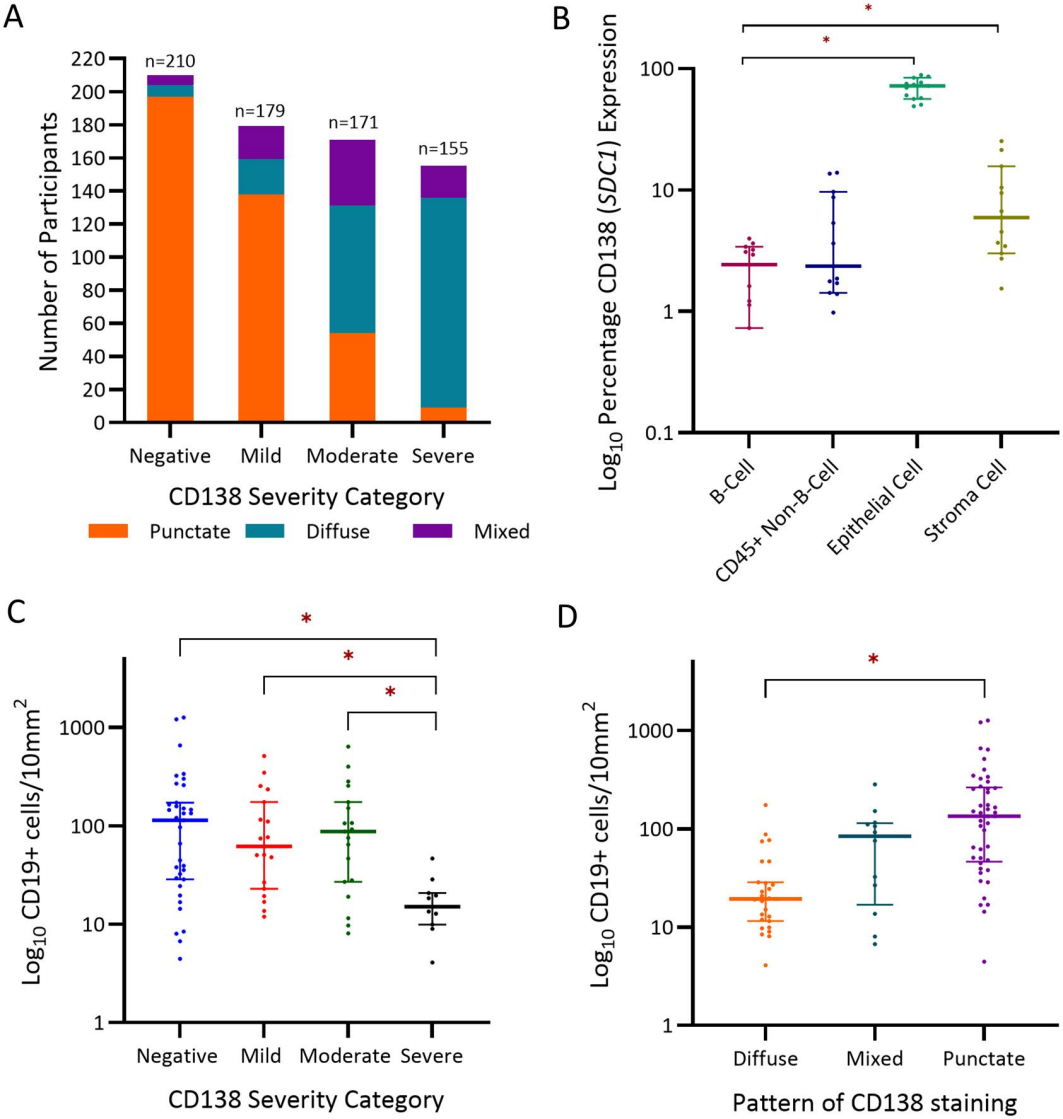
**CD138 (Syndecan-1) cell expression and immune cell overlap.** (**A**) Relationship between CD138 expression severity and pattern of CD138 expression. (**B**) CD138 (*SDC1*) is more likely to be expressed by endometrial epithelial or stromal tissue then B-Cells (Flow Cytometry Log10 Percentage Cells; *P* < 0.0001). (**C**) CD138 severity is significantly related to reduced CD19 cell density (Cells/10 mm^2^ Log10; *P* < 0.05). (**D**) CD138 expression pattern is significantly related to reduced CD19 cell density (Cells/10 mm^2^ Log10; *P* < 0.05). *Denotes significance (*P* < 0.05) on post hoc testing. SDC1, Syndecan-1/CD138.

CD138 IHC demonstrated three distinct patterns of immunostaining. These included: punctate membranous expression (55.7%; n = 398), diffuse non-membranous expression (32.4%; n = 232), and a third mixed pattern (11.9%; n = 85) (Fig. 2A). An increased proportion of diffuse immunostaining was seen with increasing CD138 severity (mild expression 10.5%, n = 19/181 vs severe expression 82.4%, n = 126/153) (Fig. 3A).

To verify the pattern of IHC staining, single-molecule in situ hybridization was performed. This demonstrated the presence of CD138 mRNA transcripts within the endometrial stroma in a similar expression pattern to that seen on IHC validating the differential expression patterns seen on IHC (Fig. 2B and C). Additionally, prominent basolateral epithelial staining was observed.

### Stromal CD138 expression is not specific to plasma cells

CD19, an alternate marker of plasma cells, immunostaining demonstrated a significant relationship between CD19 abundance and CD138 severity and expression (Fig. 3C and D). An inverse relationship was exhibited with a significantly higher CD19 abundance in CE-negative samples compared to severe CD138 immunostaining (*P* = 0.0023). Similarly, significant differential CD19 abundance in favour of punctate expression was seen in comparison to diffuse CD138 expression (*P* < 0.01). Flow cytometric analysis of 13 selected pilot endometrial biopsies demonstrated minimal overlap between CD19 and CD138 cell populations with significantly increased expression of CD138 seen in endometrial epithelial (*P* < 0.0001) and stromal populations (*P* = 0.0449) (Fig. 3B).

### Evidence for constitutive endometrial stromal CD138 expression

**Figure 4.**
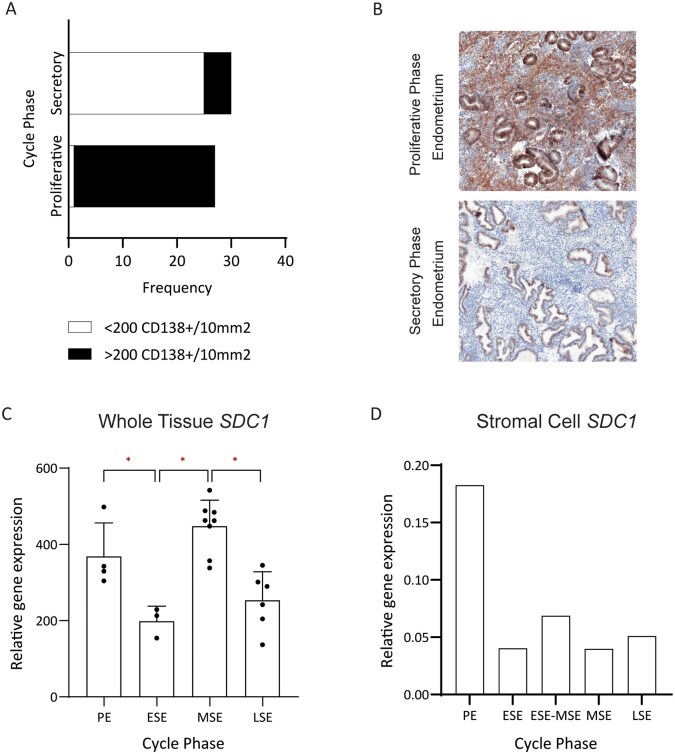
**Endometrial expression of CD138 (*SDC1*).** (**A**) Differential quantified CD138 expression in normal controls. Demonstrating a significant reduction in CD138 expression from the proliferative to secretory phase (*P* < 0.0001). (**B**) Exemplar paired proliferative phase severe CD138 stromal staining (*Upper Panel*; Brown) and secretory phase negative for stromal staining (*Lower Panel*). (**C**) Whole tissue across-cycle CD138 (*SDC1*) expression showing significant stepwise changes in expression pattern (*P* = 0.0001). Proliferative Endometrium (PE) vs Early-Secretory Endometrium (ESE) (*P* = 0.0291); ESE vs Mid-Secretory Endometrium (MSE) (*P* = 0.0004); MSE vs Late-Secretory Endometrium (LSE) (*P* = 0.0005). (**D**) Stromal cell across cycle expression of CD138 (*SDC1*). *Denotes significance (*P* < 0.05) on post hoc testing. SDC1, Syndecan-1/CD138.

Interrogation of Gene Expression Omnibus endometrial whole tissue datasets showed the presence of constitutive whole tissue *SDC1* transcript expression (Fig. 4C). A statistically significant variation in expression pattern across the menstrual cycle (one-way ANOVA; *P* = 0.0001) was noted with an initial proliferative peak observed followed by a mid-secretory peak.

Analysis of a separate published single-cell gene expression dataset collected across the menstrual cycle indicated maximum stromal expression of *SDC1* within the proliferative phase of the menstrual cycle (Fig. 4D). A reduction in relative expression was seen across the secretory phase of the menstrual cycle with a relative up-regulation in the early-mid cycle.

Cell compartment analysis revealed CD138 expression to predominate within the epithelial compartment. Clear stromal expression was however, demonstrated, with expression varying across the menstrual cycle (Supplementary Fig. S2A–C).

To explore the protein-level expression of CD138, 27 proliferative phase endometrial samples underwent CD138 IHC. In total, 26/27 (96%) samples demonstrated severe CD138 expression of >200 CD138^+^ cells per 10 mm^2^ (Fig. 4A). Comparison with a subset of secretory phase samples was performed with severity of staining dichotomized to <200/10 and >200/10 mm^2^ to accommodate the high degree of staining within the proliferative grouping. A significant reduction in the proportion of samples exhibiting severe CD138 expression across the menstrual cycle was observed (16.7% vs 96.2%; *P* < 0.01, Fisher’s exact) (Fig. 4A). Paired proliferative and secretory samples collected from the same participant further demonstrated high proliferative phase immunoreactivity which decreased in the secretory phase (Fig. 4B).

Similarly, a significant reduction in glandular epithelial CD138 expression was also seen across the proliferative to secretory phase of the menstrual cycle (mean H-score 197.51 vs 104.56; *P* < 0.05) (Supplementary Fig. S3).

### CD138-diagnosed CE is associated with an immature endometrial phenotype

**Figure 5.**
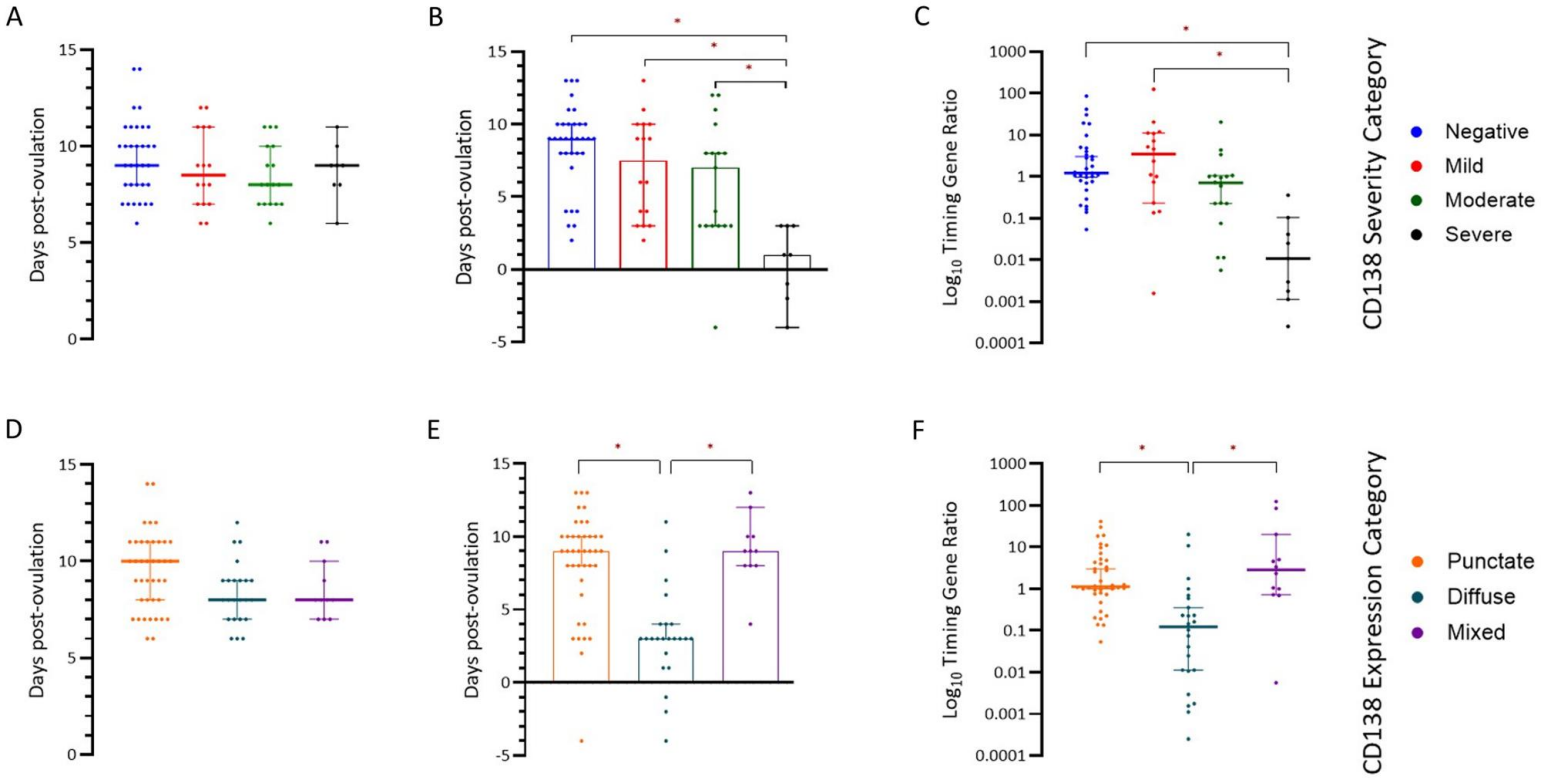
**CD138 severity is associated with delayed endometrial timing.** (**A**) Relationship between ovulation-based cycle time and CD138 severity (*P* = 0.47). (**B**) Relationship between histological time and CD138 severity (*P* < 0.0001). (**C**) Relationship between molecular timing and CD138 severity (*P* < 0.0001). (**D**) Relationship between ovulation-based cycle time and CD138 pattern (*P* = 0.07). (**E**) Relationship between histological time and CD138 pattern (*P* < 0.0001). (**F**) Relationship between molecular timing and CD138 pattern (*P* < 0.0001). *Denotes significance (*P* < 0.05) on post hoc testing.

Samples were next assessed by ovulation-based time of the cycle collected as calculated by days post-ovulatory LH surge. For comparability with later data, this analysis was performed across a cohort of 74 samples for which timing data by other methodologies was available. Samples were collected on average 8.99 ± 1.88 SD days post-ovulatory LH surge. No significant difference in CD138 expression severity (*P* = 0.47) or expression pattern (*P* = 0.07) was seen when analysed by patient-reported day of the cycle collected (Fig. 5A and D).

Histological timing of endometrial samples was undertaken utilizing Noye’s criteria. A significant difference in histological time between groups was seen when assessed by CD138 expression severity (Kruskal–Wallis: *P* < 0.01). Post hoc analysis demonstrated this effect to be driven by a significant decrease in histological cycle day in the severe group, mean day of cycle post-LH surge 0.5 compared to Day 8.5 for negative severity expression (*P* < 0.01); Day 7 for mild expression (*P* = 0.01) and Day 6 for moderate expression (*P* = 0.04) (Fig. 5B).

Significant across-group differences in histological time were seen by pattern of CD138 expression (Kruskal–Wallis: *P* < 0.01). A significantly lower histological day of cycle was seen in the diffuse expression type with a mean of 3.1 days post-LH surge compared to 8.2 days in samples with punctate expression (*P* < 0.01) and 9.1 days in mixed expression pattern (*P* < 0.01) (Fig. 5E).

Further, a modified molecular timing method was used based on a previously published methodology (Lipecki *et al.*, 2022). A significantly lower ratio was seen in severe CD138 expression in comparison to negative samples (Median Ratio 0.011 vs 1.202; *P* < 0.01) correlating with an earlier histological time (Fig. 5C). Similarly, a reduction in timing ratio in diffuse CD138 expression in comparison to punctate or mixed expression was demonstrated, median ratio 0.123 vs 1.127 and 2.828, respectively; *P* < 0.01 and *P* = 0.05 (Fig. 5F).

### Assessment of Tao brush sampling for microbiota profiling of the endometrium

For metataxonomic analysis, 50 samples from across the reproductive tract (vagina, ectocervix, endocervical canal, endometrial tissue, and endometrial sampling with a Tao brush device) were acquired from 10 women. DNA was extracted and samples sequenced yielding a total of 2 778 602 read counts across the whole dataset (Supplementary Fig. S4A). Endometrial sampling with the Tao brush yielded a median number of reads of 60 795 (Min–Max: 39 886–76 420), comparable to vaginal swabs (Median: 51 064, Min–Max: 25 896–63 639) or endometrial tissue biopsy (Median: 47 724 Min–Max: 26 848–60 027). Three endocervical and three ectocervical samples produced <5000 reads, likely due to a low bacterial load. Negative control produced on average 390 read counts/samples suggesting minimal environmental or processing contamination (Supplementary Fig. S4B). The number of ASVs and Shannon α-diversity index observed were also higher for Tao brush, endometrial tissue biopsy, and vaginal swab compared to their corresponding negative controls (Supplementary Fig. S4C and D).

### The endometrial microbiome represents a continuum of the lower reproductive tract

Matched vaginal, ectocervical, and endometrial Tao brush samples were collected from 114 patients. Hierarchical clustering analysis of the endometrial and ectocervical samples identified clustering patterns similar to those described in the literature for the vaginal mucosa (Supplementary Figs S5, S6, S7, and S8). β-diversity, with PERMANOVA analyses also indicated that sampling site explained only a small proportion of variance in the dataset (*R*^2^ = 0.0154, *P *< 0.001). Owing to the similarity between endometrial and vaginal metataxonomic profiles, we applied the VALENCIA CST classifier to the endometrial samples (Supplementary Fig. S5). The prevalence of CSTs and their concordance (Fleiss’ κ = 0.94, *P *< 0.05) within individuals across the different anatomical sites (Supplementary Fig. S9) suggests an interconnected microbial ecosystem rather than isolated niche communities.

A series of statistical analyses was also conducted to assess whether reproductive microbiome profiles varied with cycle timing in the luteal phase. These included multinomial logistic regressions using the full CST categories as outcome (CST I, II, III, IV-B, IV-C, and V), logistic regressions with a simplified *Lactobacillus* dominant (CST = I, II, III, or V) vs *Lactobacillus* depleted community (CST IV-B, or IV-C) outcome, regression analyses of the centred-log-ratio-transformed counts of species-level taxa against cycle timing measures, and linear regression models between Shannon α-diversity and timing measures. These analyses were performed also for ectocervical and vaginal profiles. With the exception of an association between endometrial Shannon α-diversity and the reported luteal cycle timing (one-way ANOVA *P*-value = 0.041), which upon visual inspection of scatterplots appears spurious (Supplementary Fig. S10), no statistically significant associations were found between any of these microbial community composition descriptors and timing measures.

### CE-positive endometrium is not associated with an altered endometrial or vaginal microbiota

**Figure 6.**
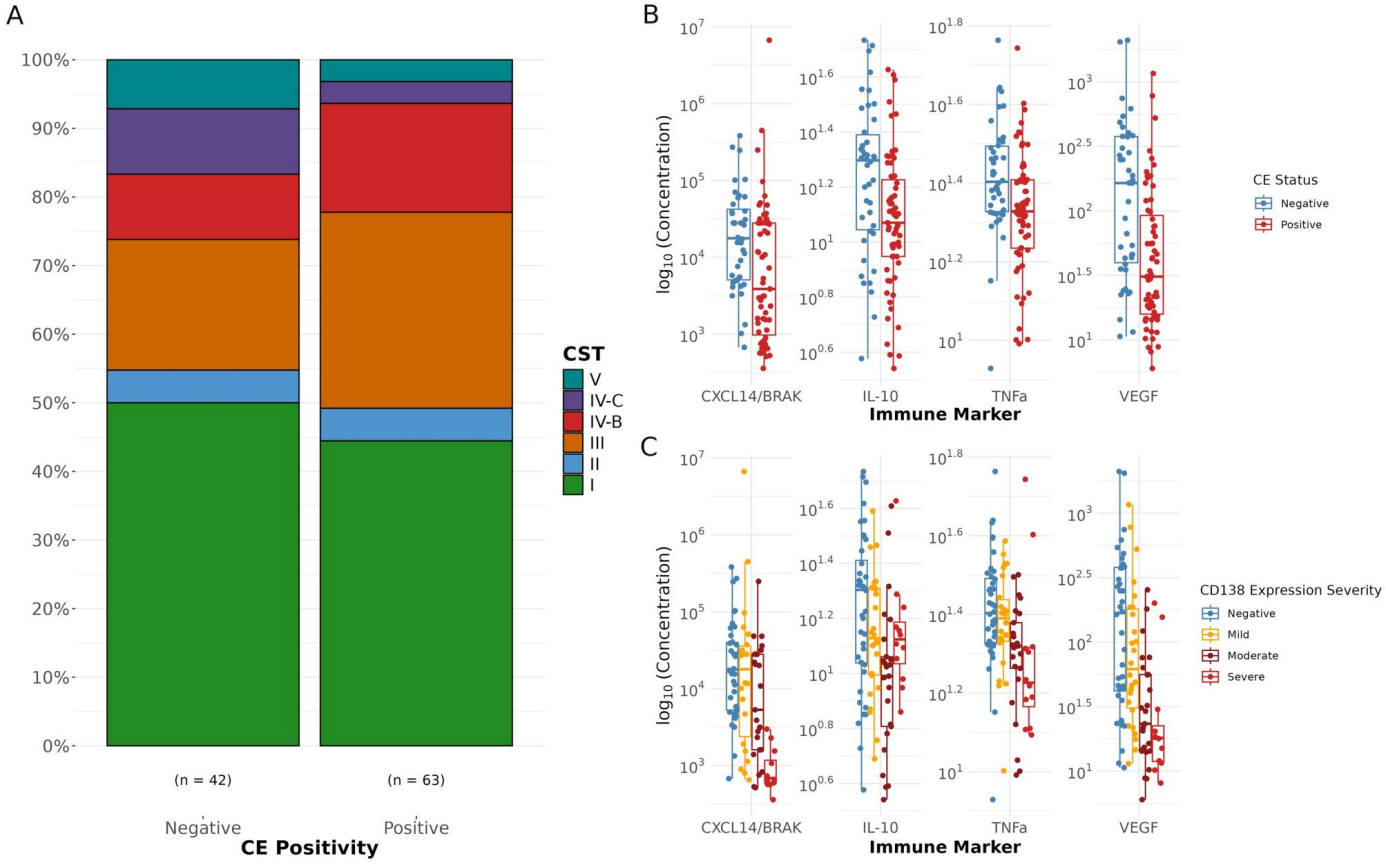
**CD138 expression and severity is associated with inflammatory but not microbiome alterations** (**A**) Distribution of endometrial CSTs in CE-positive and CE-negative individuals. Relationship between immune marker levels and CE positivity (**B**) and expression severity. (**C**) Shown only for analytes with statistically significant differences (*q*-value < 0.05). CST, community state types; CE, chronic endometritis.

No significant differences in prevalence of vaginal, ectocervical, or endometrial CST was demonstrated in those with CE in comparison to those without (Fig. 6A, Supplementary Fig. S11). CD138 expression pattern and severity also did not show any association with CST. Differential relative abundance analyses also showed no association between CD138 diagnostic, severity, or expression pattern severity and specific species-level or taxon. This was consistent across all three sampling sites.

### CE-positive endometrium has an altered inflammatory cytokine profile

To assess if CD138 could be associated with local immune response, CXCL14, IL-10, IL-15, IL-6, IL-8, SLPI, TNF-α, and VEGF were quantified in vaginal and endometrial samples. Statistically significant differences (*q *< 0.05, type II ANOVA test of linear regression coefficient with *Benjamini–Hochberg* FDR correction) were identified for endometrial levels of CXCL14, IL-10, TNF-α, and VEGF between those with CE compared to those without (Fig. 6B). These markers also displayed a statistically significant relationship with CD138 expression severity (*q* < 0.05, Fig. 6C). Except for IL-10, these also showed differences in abundance levels with CD138 expression pattern (*q *< 0.05, Supplementary Fig. S12). No significant associations with CE positivity, expression pattern, or severity were identified in vaginal soluble immune marker concentrations. None of these markers displayed any statistically significant association with the CST from the same anatomical location. However, three of the four immune mediators reported to differ with CE diagnosis were also found to positively correlate with cycle timing. Endometrial concentrations of CXCL14/BRAK, TNFα, and VEGF were associated with luteal phase timing assessed histologically (linear regression one-way ANCOVA, BH FDR *q*-value < 0.05, Supplementary Fig. S13A). The same trends were observed with the reported luteal phase timing, but only for CXCL14/BRAK and VEGF. Instead, IL-15 was found to also withstand multiple testing correction (*q*-value < 0.05, Supplementary Fig. S13B) but the trend visualized in the scatterplot seems weaker and potentially spurious (Supplementary Fig. S13B). For the molecular timing ratio, CXCL14/BRAK, TNFα, VEGF, and IL-8 were also found to have a statistically significant association (*q*-value < 0.05) (Supplementary Fig. S14). No associations were found between timing parameters and vaginal concentrations of immune mediators.

## Discussion

CD138-diagnosed CE is an emergent area of clinical interest in RPL. Despite this, the pathophysiology of CE remains poorly elucidated. This study sought to determine the constitutive expression of CD138 within the endometrial stroma and its relationship with the pathogenesis of RPL and specifically, composition of the reproductive tract microbiota.

While previous studies have reported on the wide prevalence range of CD138^+^ CE (Liu *et al.*, 2018), our study demonstrates three key differential immunostaining patterns of stromal CD138. It is clear based on the wide range of prevalence across studies that differential approaches to inclusion of these staining patterns have occurred. This study utilized previously published diagnostic methodology which has been demonstrated to be associated with reduced inter- and intra-observer variability in addition to correlating with clinical outcome (Liu *et al.*, 2018). This more permissive definition of CE has provided the scope to analyse for effect based both on the staining pattern, which is likely differentially included by groups and the severity, encompassing the range of definitions utilized within the literature. This approach contrasts with the majority of studies within the literature which underreport on differential staining characteristics seen and utilize research group specific-diagnostic criterion without clinical correlation for untreated effect (Reig, 2025). This has likely resulted in the lower prevalence seen in some studies but also the loss of resolution on better understanding the potential pathophysiology of stromal CD138 expression. By following a CD138 expression-based approach, this study has sought to interrogate the endometrium across the range of definitions seen. A further recent high-quality study has replicated this severity stratification model (De Smet *et al.*, 2024).

Multi-modal interrogation of the endometrium demonstrated a cyclical pattern of expression with upregulation within the proliferative phase of the menstrual cycle. Lai *et al.* (2007) reported similar findings but our study is the first to demonstrate both transcription and protein-level changes in CD138 across the cycle. Our findings highlight the importance of standardization of timing in CD138-based CE diagnosis.

Further confirmation of this immune-independent CD138 expression was demonstrated utilizing flow cytometry, which failed to show overlap between CD19-gated and CD138-gated cell populations. Furthermore, an inverse relationship between CD138 severity and CD19 was demonstrated. CD19 is an alternate cell membrane-based marker of plasma cells. This marker is often utilized in non-CE based lymphocyte analysis and has been widely demonstrated to be highly expressed by plasma cells (Lee *et al.*, 2021; Tian *et al.*, 2023). Lack of population overlap therefore demonstrates the likely inclusion of non-plasma cell-based CD138+ cells within currently used diagnostic criteria. Consistent with these findings, a recent study assessing 80 women undergoing IVF reported that 49% had ≥1 plasma cell/10 hpf with no impact on clinical pregnancy, pregnancy loss, or live birth (Herlihy *et al.*, 2022). Further, (McQueen *et al.*, 2021) reported that 31% of control cases in a study assessing women with RPL also demonstrated one or more plasma cells within the endometrial stroma. Both studies utilizing CD138 IHC highlighting this likely non-immune inclusion.

Despite its constitutive expression, several studies utilizing CD138-based diagnostic criterion have demonstrated an association with both reduced live birth rates and higher rates of miscarriage (McQueen *et al.*, 2015; Tersoglio *et al.*, 2015; Zhang *et al.*, 2019). This association together with the data from our study suggests that this relationship may be driven by a non-immune-based mechanism of pregnancy loss. Our study has demonstrated a cyclical pattern to CD138 expression. Persistence of CD138 into the secretory phase of the menstrual cycle may represent an aberrant across-cycle maturation of the endometrium. This is demonstrated by the histological and transcriptional immaturity of CD138-enriched endometrial tissue shown within this study. Failure of endometrial progression across the menstrual cycle has previously been shown to be associated with RPL, further reinforcing this hypothesis (Lipecki *et al.*, 2022). This would suggest a potential mechanism for the CD138-linked adverse pregnancy outcome seen in associative studies of CE. It is possible that the association seen is driven by either CE-mediated abnormal endometrial development or, alternatively, through CD138 expression as a confounder, linking immature endometrium and adverse pregnancy outcome. The latter is reinforced by the lack of immune association demonstrated in our study.

Several studies have reported a potential link between CE and pathogenic or dysbiotic bacterial colonization of the endometrium (Cicinelli *et al.*, 2014; Kitaya *et al.*, 2017; Liu *et al.*, 2019). Using careful sampling methods and accounting for different sources of contamination, we found no association between CE and microbial composition across differing reproductive tract sites. This is in contrast to Liu *et al.* (2019) who demonstrated a reduction in Lactobacilli species in those with CE, with an increased relative abundance of non-Lactobacilli species including those of Bifidobacterium and Prevotella. These differences may be partly explained by differences in sampling methodology, with Liu *et al.* (2019) utilizing an endometrial fluid-based lavage methodology as opposed to our tissue surface sampling. The lavage methodology poses high risk of cross-contamination with lower reproductive tract taxa and although the study incorporated negative controls it was unclear how these were accounted for within the analyses. It remains therefore plausible that tissue-based sampling methodology may be more likely to reflect the incumbent resident microbial population of the endometrium. In addition, no significant relationship was observed between different microbial profiles or CSTs and CE. CST classifications were determined on the basis of the dominant bacterial species and overall composition within each sample (France *et al.*, 2020; Parraga-Leo *et al.*, 2024). For example, CST-I represents those samples dominated by *Lactobacillus crispatus*. Despite not observing a link between microbiota and CD138-based CE diagnosis, our results showed that a positive diagnosis was associated with a disordered endometrial cytokine profile. A reduction in expression of CXCL14, IL-10, TNF-α, and VEGF was seen in CD138+ endometrium. These findings are consistent with previous work which demonstrated reduced IL-10 expression in CE-positive endometrium (Wang *et al.*, 2019). Secretion of IL-10 is partly driven by T-helper cells 2 (TH2). Alterations in the balance of TH1 and TH2 secreted cytokines have previously been associated with RPL (Kwak-Kim *et al.*, 2003). Of note, this imbalance is driven by both IL-10 downregulation and upregulation of TNF-α, an effect not seen in this study. However, we also observed temporal associations between cycle timing and CXCL14, TNF-α, and VEGF. This suggests that rather than being immune-driven, these changes are driven by endometrial immaturity. Poor proliferative phase expression of TNF-α has been shown to correlate with evidence of immaturity (Philippeaux and Piguet, 1993). This expressional immaturity may contribute to poor implantation pre-disposing to miscarriage (You *et al.*, 2021). Similarly, down-regulation of VEGF has been associated with RPL (Abu-Ghazaleh *et al.*, 2023). Across-cycle analysis of VEGF expression demonstrated altered temporal expression however, this was inconsistent across the cycle suggesting this finding to be driven by endometrial immaturity rather than mistiming (Lai *et al.*, 2015).

Collectively, our findings suggest that the association between RPL and CD138^+^ CE may be driven primarily by endometrial immaturity rather than immune-driven differential endometrial changes. Given this, antibiotic-based treatment is unlikely to be effective and a luteal-phase supplementation strategy may be more plausible. This, however, remains to be assessed in clinical randomized controlled studies.

### Strengths and limitations

This study provides the most detailed assessment to date of microbiota-immune interactions in the endometrium in women with CE, as defined by CD138 expression. Integration of a cohort-based dataset with publicly available datasets highlights consistency in findings across these different data sources. The study was conducted prospectively with prior publication of the protocol (Odendaal *et al.*, 2023). Limitations of the study include the use of multiple statistical testing. This was mitigated by concurrence of findings across different data types. These were performed in the absence of powering given the exploratory nature of the work and its association with the clinical trial which was powered to clinical outcomes. Nevertheless, this is mitigated by the significant finding of endometrial immaturity and the totality of evidence across different modalities.

The key finding of disordered endometrial timing in this population relied on patient-reported ovulation-based timing. It is recognized that corollary serum progesterone levels would have further strengthened this finding. Despite this, the strong effect seen suggests this as an unlikely source of bias in the study.

Additionally, a higher prevalence of CD138 was noted within our population in comparison to previously reported studies. This reflects the meticulous methodology of this study which utilized published methodology aligned with the automated clinical pathology process, made use of second observers and was conducted within an enriched population. This aligns with the literature which has demonstrated a broad diagnostic criterion to be associated with a higher incidence of CE (OR 2.96; 95% CI 1.13–6.44) (Huang *et al.*, 2020). These findings likely stem from the inclusion by different groups of differential staining patterns. To mitigate for this, a subgroup analysis by both CD138 severity and staining pattern was performed within this study. It is notable that a similar approach is now also being used by emergent studies (De Smet *et al.*, 2024). This inclusive approach with a high prevalence has strengthened this study’s conclusions allowing the demonstration of a biological gradient and differential characteristics associated with CD138 staining patterns. Finally, the study is limited by lack of collection of data on underlying fertility-related co-morbidities. This was secondary to exclusion of participants at the study recruitment stage where an existing co-morbidity was contributory to their pregnancy loss but it remains possible that non-contributory diagnoses are present.

## Conclusion

This study demonstrates that CD138^+^ endometrial expression is associated with impaired endometrial timing driven by a failure of endometrial maturation across the menstrual cycle, rather than immune or microbiota changes in those with CD138^+^ CE. Thus, antibiotic-based treatment strategies for CE are unlikely to be effective. CE testing should therefore not be performed outside of a research context while correlation of these findings with clinical outcomes is performed.

## Supplementary Material

deag032_Supplementary_Figure_S1

deag032_Supplementary_Figure_S2

deag032_Supplementary_Figure_S3

deag032_Supplementary_Figure_S4

deag032_Supplementary_Figure_S5

deag032_Supplementary_Figure_S6

deag032_Supplementary_Figure_S7

deag032_Supplementary_Figure_S8

deag032_Supplementary_Figure_S9

deag032_Supplementary_Figure_S10

deag032_Supplementary_Figure_S11

deag032_Supplementary_Figure_S12

deag032_Supplementary_Figure_S13

deag032_Supplementary_Figure_S14

deag032_Supplementary_Table_S1

## Data Availability

Anonymized participant data can be requested by contacting the data access team in the Warwick CTU wctudataaccess@warwick.ac.uk. Microbial and Metataxonomic raw data are available in the European Nucleotide Archive (Projects PRJEB83331 and PRJEB83332). Scripts and coding used for data preprocessing are available in the Github repository supporting this publication (https://github.com/Gscorreia89/cerm-ce).
