# Supplementary figures and images for: CD138 expression in the endometrium associates with endometrial timing and inflammatory status but not microbiota composition
Source: Hum Reprod. 2026 Mar 20;41(5):699–711. doi: 10.1093/humrep/deag032 (PMC13139656; doi:10.1093/humrep/deag032)

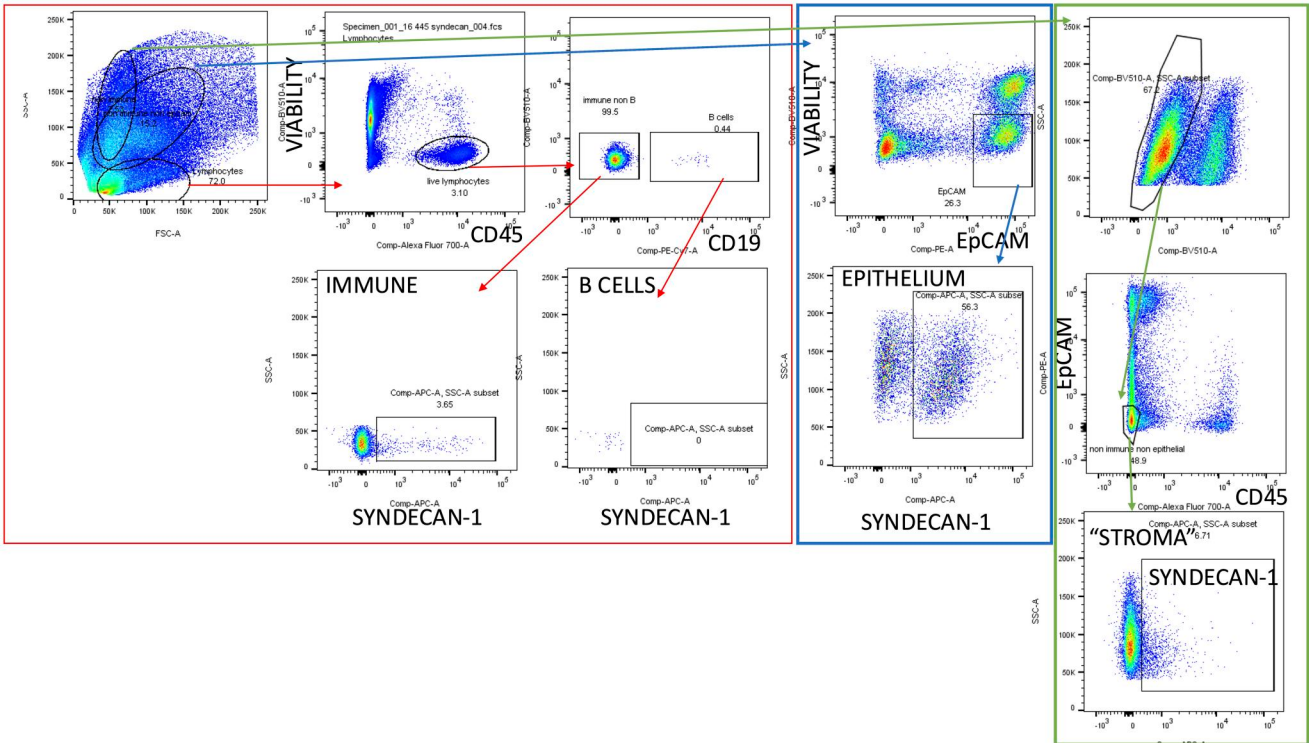

Supplementary Figure S1. Flow cytometry gating strategy.

Supplement: deag032_Supplementary_Figure_S1 [file deag032_supplementary_figure_s1.pdf]
