# Supplementary material for: CD138 expression in the endometrium associates with endometrial timing and inflammatory status but not microbiota composition
Source: Hum Reprod. 2026 Mar 20;41(5):699–711. doi: 10.1093/humrep/deag032 (PMC13139656; doi:10.1093/humrep/deag032)
Supplement: deag032_Supplementary_Figure_S2 [file deag032_supplementary_figure_s2.pdf]

OXFORD

human reproduction

Human Reproduction, 2026, 1–1

https://doi.org/10.1093/humrep/deag032

Supplementary Data

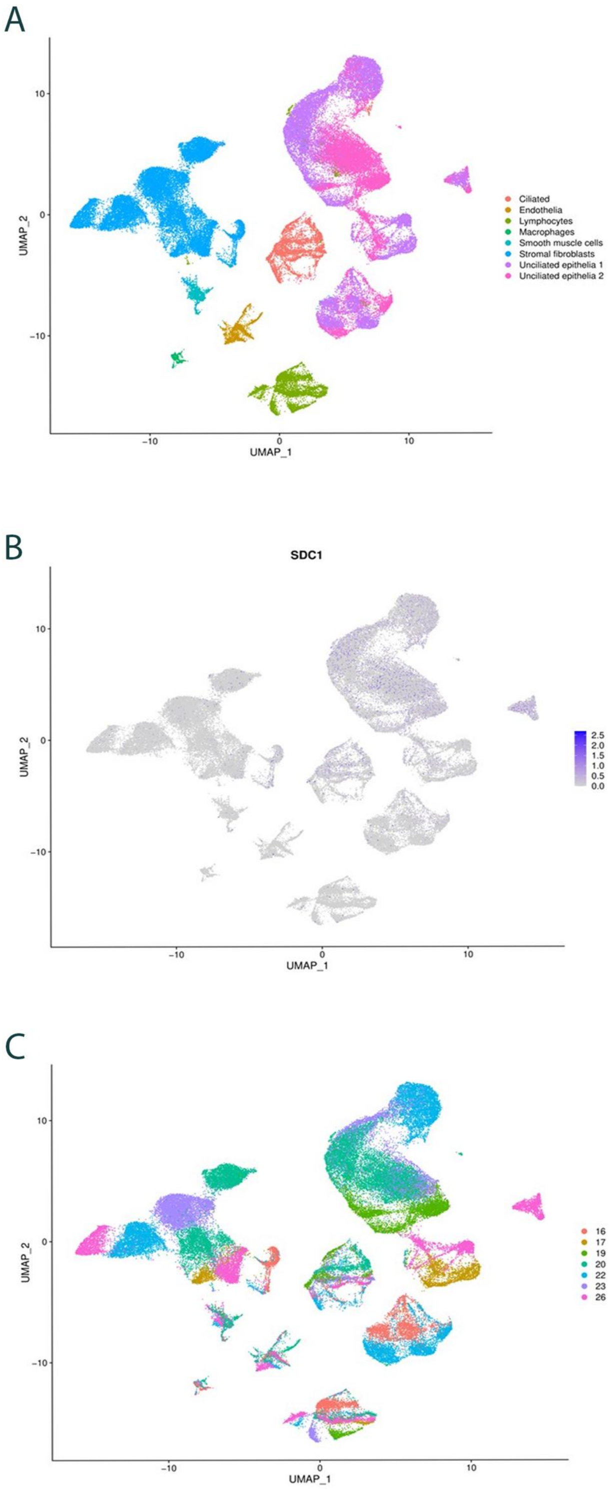

Supplementary Figure S2. Single-cell sequenced spatial distribution. (**A**) Endometrial cell types demonstrating differentiation of stromal cells, epithelial cells, and immune cells with their respective sub-types. (**B**) Distribution of SDC1 across the endometrium. Purple dots indicate cells expressing SDC1. SDC1 is demonstrated across a range of cell types notably epithelial cells but in addition stromal cells. SDC1, Syndecan-1/CD138. (**C**) Temporal distribution of cells demonstrating cellular groupings across Days 16, 17, 18, 19, 20, 22, 23, 26 of the cycle.
