# Supplementary material for: CD138 expression in the endometrium associates with endometrial timing and inflammatory status but not microbiota composition
Source: Hum Reprod. 2026 Mar 20;41(5):699–711. doi: 10.1093/humrep/deag032 (PMC13139656; doi:10.1093/humrep/deag032)
Supplement: deag032_Supplementary_Figure_S3 [file deag032_supplementary_figure_s3.pdf]

Human Reproduction, 2026, 1–1
https://doi.org/10.1093/humrep/deag032
Supplementary Data

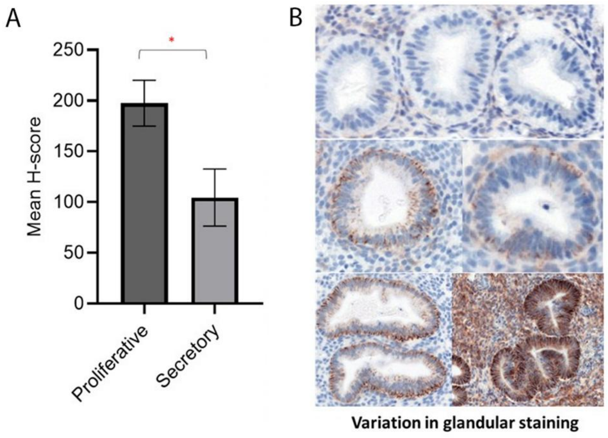

Supplementary Figure S3. Glandular epithelial CD138. (**A**) Reduction in glandular epithelial CD138 across menstrual phases—mean H-score 197.51 vs 104.56; $P < 0.05$. (**B**) Exemplars of differential CD138 staining strength within the glandular epithelium showing the variation in strength seen. *Denotes $P < 0.05$.
