# Supplementary material for: CD138 expression in the endometrium associates with endometrial timing and inflammatory status but not microbiota composition
Source: Hum Reprod. 2026 Mar 20;41(5):699–711. doi: 10.1093/humrep/deag032 (PMC13139656; doi:10.1093/humrep/deag032)
Supplement: deag032_Supplementary_Figure_S4 [file deag032_supplementary_figure_s4.pdf]

OXFORD

human reproduction

Human Reproduction, 2026, 1–1
https://doi.org/10.1093/humrep/deag032
Supplementary Data

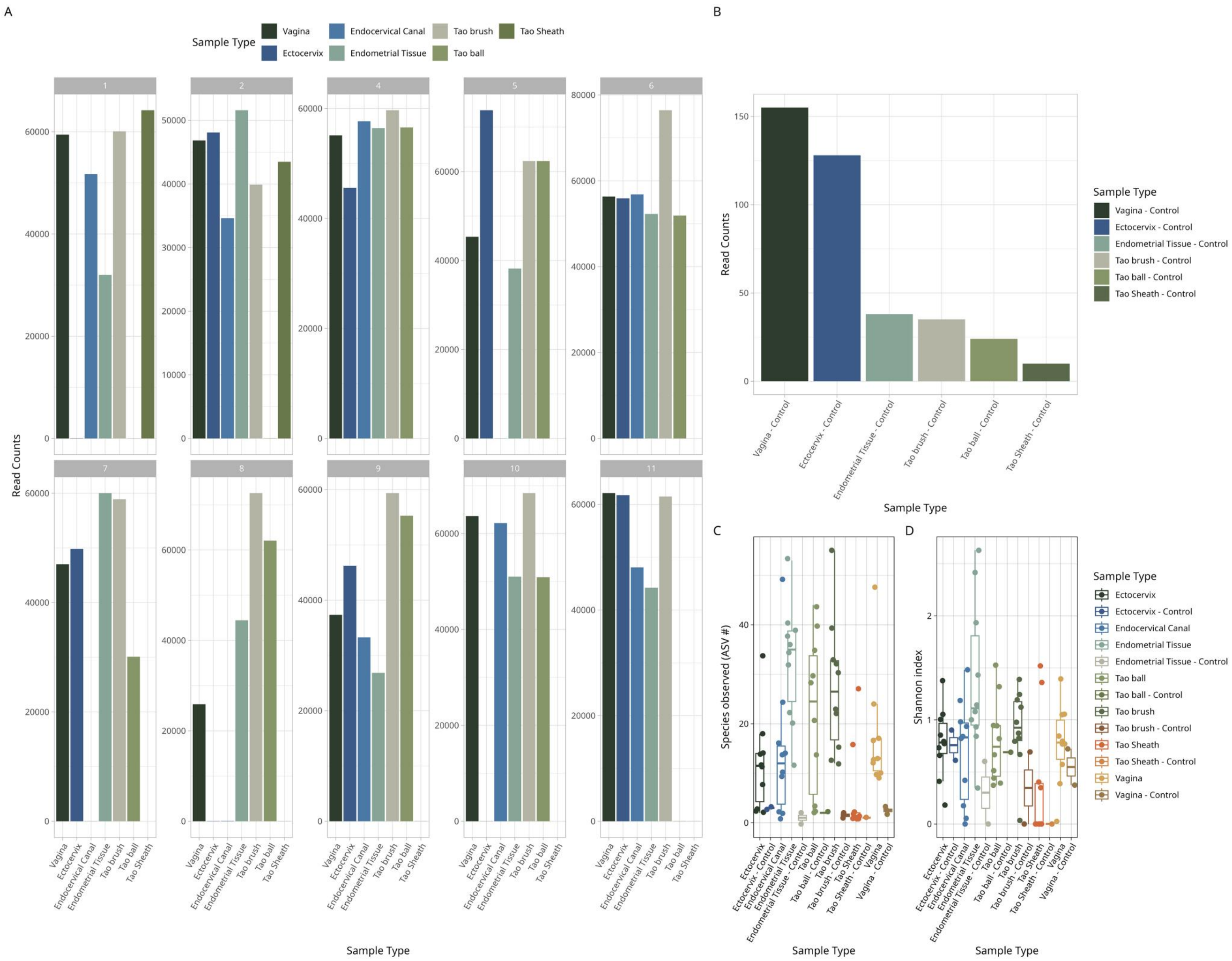

Supplementary Figure S4. Summary of results from the pilot sampling test. (**A**) Total number of reads for each sample type (including controls), facetted by individual. (**B**) Total number of read obtained from the negative controls. (**C**) Number of species observed per sample type. (**D**) Shannon α-diversity index per sample type.
