# Supplementary material for: CD138 expression in the endometrium associates with endometrial timing and inflammatory status but not microbiota composition
Source: Hum Reprod. 2026 Mar 20;41(5):699–711. doi: 10.1093/humrep/deag032 (PMC13139656; doi:10.1093/humrep/deag032)
Supplement: deag032_Supplementary_Figure_S5 [file deag032_supplementary_figure_s5.pdf]

Human Reproduction, 2026, 1–1
https://doi.org/10.1093/humrep/deag032
Supplementary Data

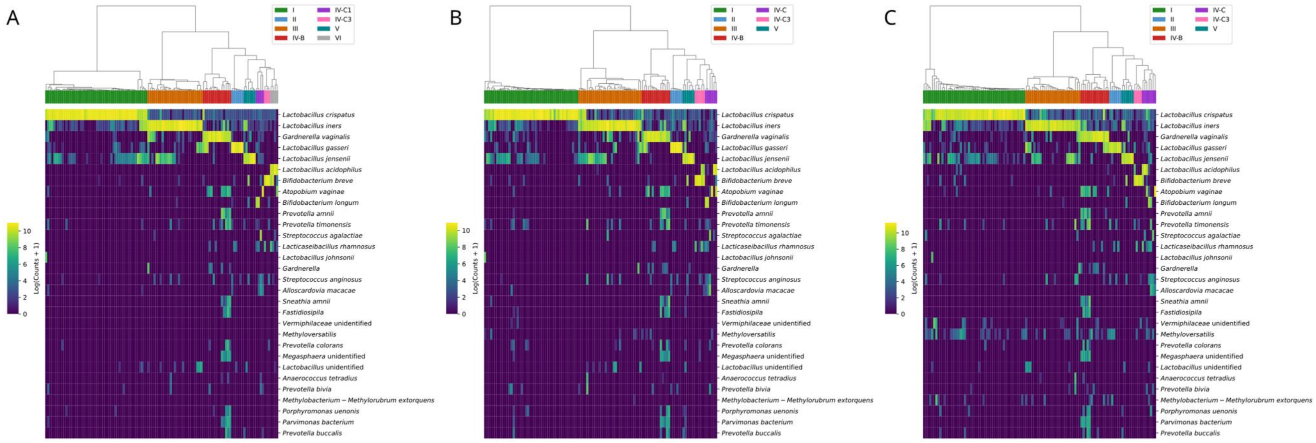

Supplementary Figure S5. Hierarchical clustering-dendrograms and heatmaps (of log-transformed taxon counts) from the vaginal (A), ectocervical (B), and endometrial (C) metataxonomic profiles. Clusters where manually labelled according to their similarity to 'VALENCIA' CSTs.
