# Supplementary material for: CD138 expression in the endometrium associates with endometrial timing and inflammatory status but not microbiota composition
Source: Hum Reprod. 2026 Mar 20;41(5):699–711. doi: 10.1093/humrep/deag032 (PMC13139656; doi:10.1093/humrep/deag032)
Supplement: deag032_Supplementary_Figure_S7 [file deag032_supplementary_figure_s7.pdf]

Human Reproduction, 2026, 1–1
https://doi.org/10.1093/humrep/deag032
Supplementary Data

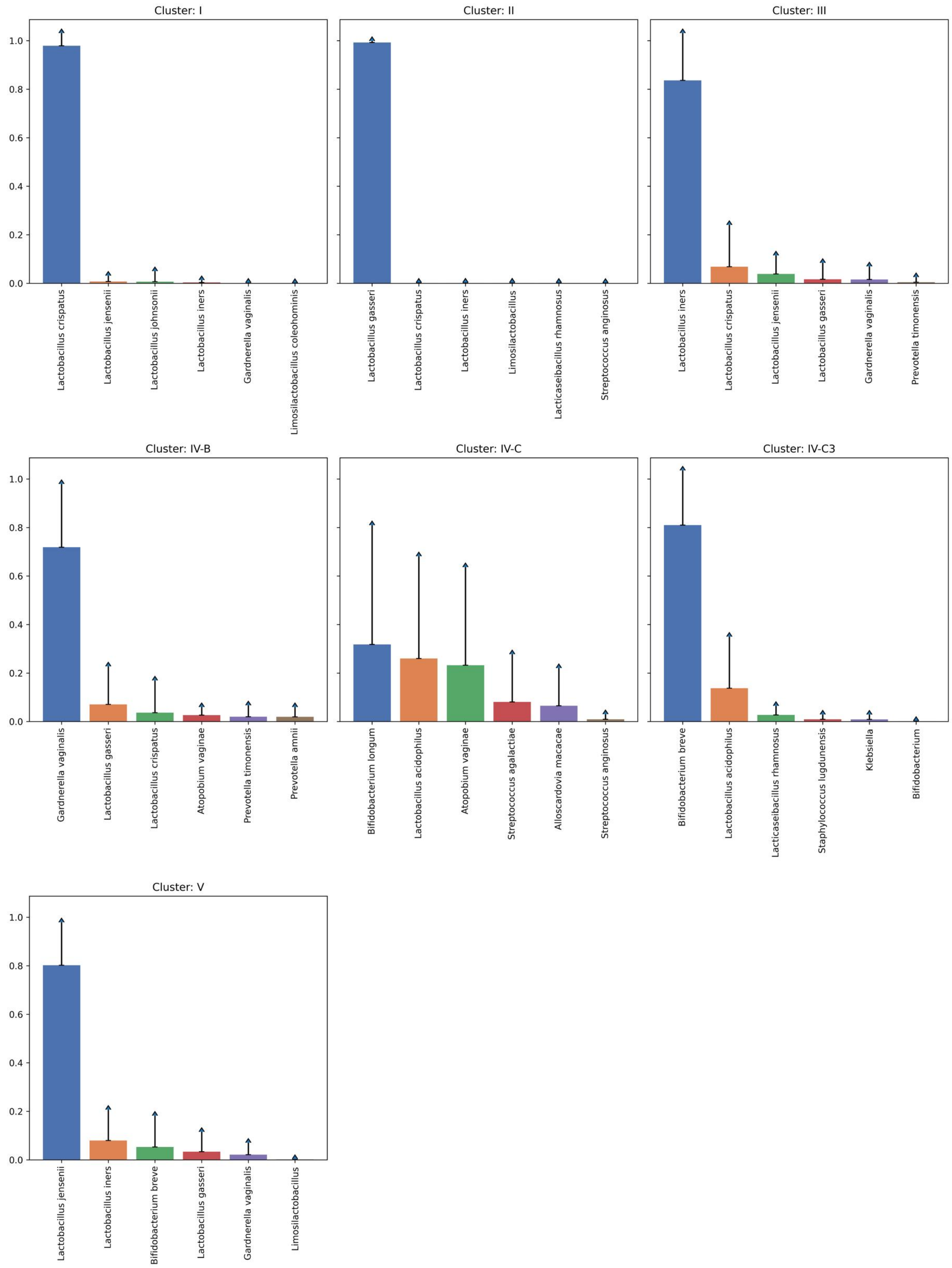

Supplementary Figure S7. Top 5 taxa by relative abundance for each of the clusters identified in the hierarchical clustering analysis of the ectocervical metataxonomic profiles. Clusters where manually labelled according to their similarity to 'VALENCIA' CSTs.
