# Supplementary material for: CD138 expression in the endometrium associates with endometrial timing and inflammatory status but not microbiota composition
Source: Hum Reprod. 2026 Mar 20;41(5):699–711. doi: 10.1093/humrep/deag032 (PMC13139656; doi:10.1093/humrep/deag032)
Supplement: deag032_Supplementary_Figure_S9 [file deag032_supplementary_figure_s9.pdf]

OXFORD
human reproduction
Human Reproduction, 2026, 1–1
https://doi.org/10.1093/humrep/deag032
Supplementary Data

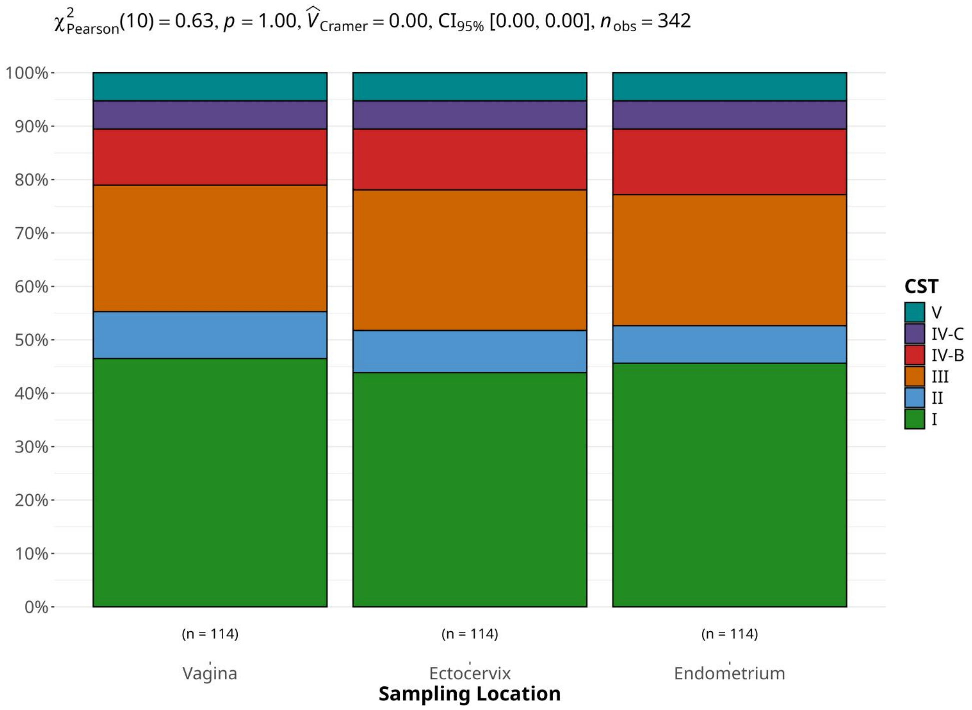

Supplementary Figure S9. Distribution of bacterial community state types in each of the anatomical locations sampled. CST prevalence was not found to differ between anatomical locations (Pearson's χ^2 test *P*-value = 0.63).
