# Supplementary material for: CD138 expression in the endometrium associates with endometrial timing and inflammatory status but not microbiota composition
Source: Hum Reprod. 2026 Mar 20;41(5):699–711. doi: 10.1093/humrep/deag032 (PMC13139656; doi:10.1093/humrep/deag032)
Supplement: deag032_Supplementary_Figure_S10 [file deag032_supplementary_figure_s10.pdf]

Human Reproduction, 2026, 1–1
https://doi.org/10.1093/humrep/deag032
Supplementary Data

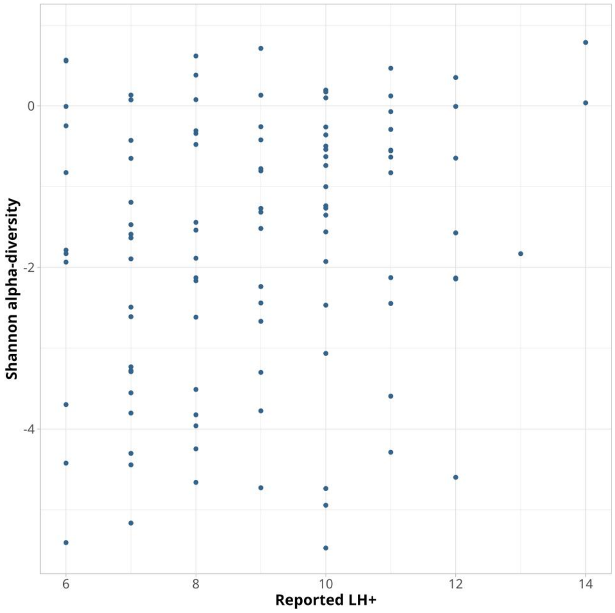

Supplementary Figure S10. Distribution of community state types with chronic endometritis status in vaginal (A), ectocervical (B), and endometrial (C) samples. No statistically significant differences were found between CE negative and positive cases in any of the sampling sites (Pearson's χ^2 test *P*-value > 0.05).
