# Supplementary material for: CD138 expression in the endometrium associates with endometrial timing and inflammatory status but not microbiota composition
Source: Hum Reprod. 2026 Mar 20;41(5):699–711. doi: 10.1093/humrep/deag032 (PMC13139656; doi:10.1093/humrep/deag032)
Supplement: deag032_Supplementary_Figure_S12 [file deag032_supplementary_figure_s12.pdf]

OXFORD
human reproduction
*Human Reproduction*, 2026, 1–1
https://doi.org/10.1093/humrep/deag032
Supplementary Data

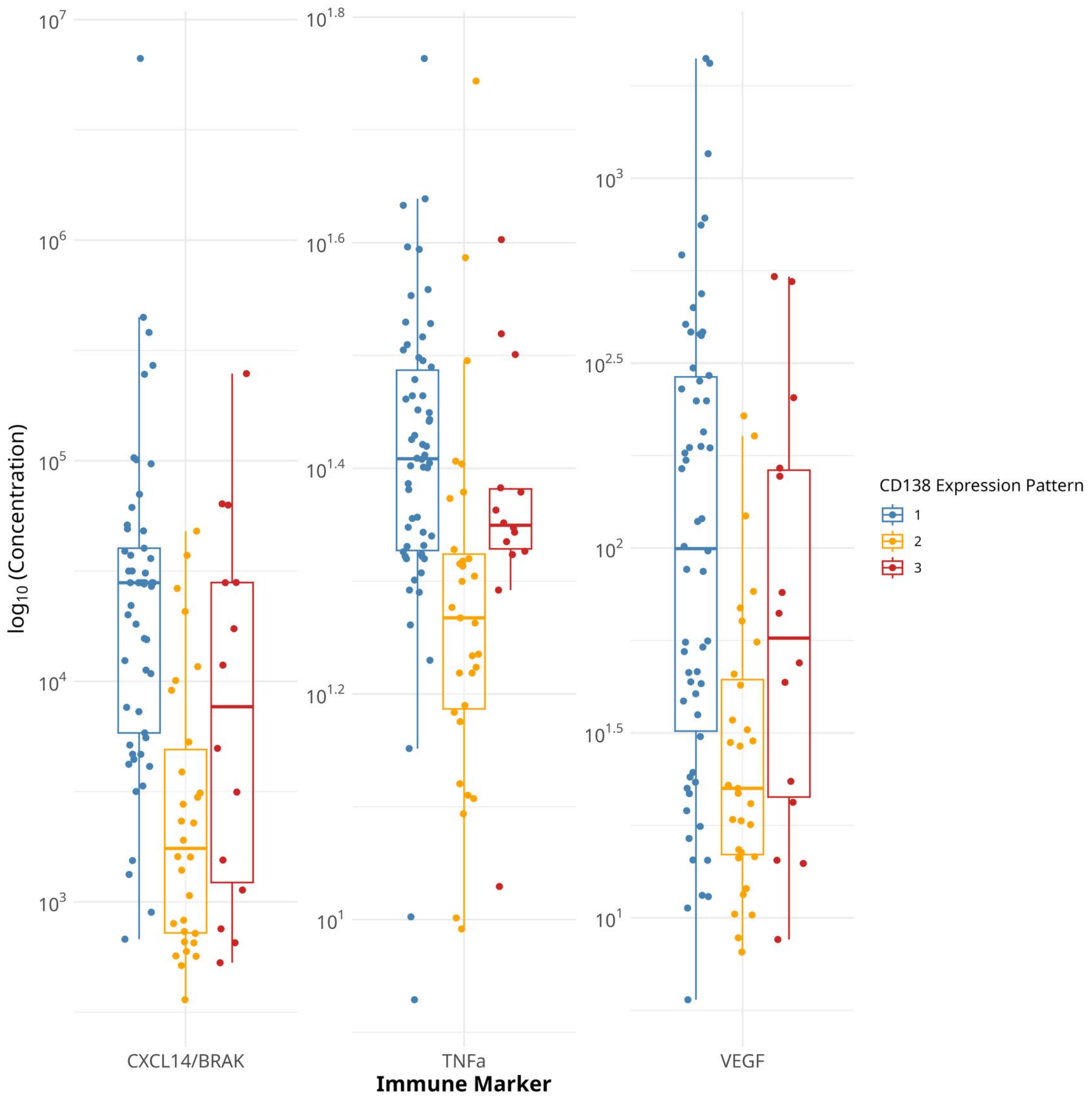


**Supplementary Figure S12.** Relationship between CXCL14/BRAK, TNF-α, and VEGF concentrations with CD138 expression pattern.
