# Supplementary material for: CD138 expression in the endometrium associates with endometrial timing and inflammatory status but not microbiota composition
Source: Hum Reprod. 2026 Mar 20;41(5):699–711. doi: 10.1093/humrep/deag032 (PMC13139656; doi:10.1093/humrep/deag032)
Supplement: deag032_Supplementary_Table_S1 [file deag032_supplementary_table_s1.pdf]

Human Reproduction, 2026, 1–1
https://doi.org/10.1093/humrep/deag032
Supplementary Data

Supplementary Table S1. Primer sequences.

Primer	Forward/Reverse	Sequence (5′-)
**L19**	Forward	GCGGAAGGGTACAGCCAAT
	Reverse	GCAGCCGGCGCAAA
**GPX3**	Forward	GGGGACAAGAGAAGTCGAAGA
	Reverse	TGTCTCCTGCCTTGGCTAAAC
**SLC15A2**	Forward	AGGAGGCATCAAACCCTGT
	Reverse	CTAGTCCGTTCCTCTGCATGT
**SCARA5**	Forward	CATGCGTGGGTTCAAAGGTG
	Reverse	CCATTCACCAGGCGGATCAT
**DIO2**	Forward	ACTCGGTCATTCTGCTCAA
	Reverse	TTCCAGACGCAGCGCAGT
**16S rRNA**	Forward	GCCTTGCCAGCCCGCTCAGTCAGAGTTTGATCCTGGCTCAG
	Reverse	GCCTCCCTCGCGCCATCAGACACACTGCATGCTGCCTCCCGTAGGAGT

© The Author(s) 2026. Published by Oxford University Press on behalf of European Society of Human Reproduction and Embryology.
This is an Open Access article distributed under the terms of the Creative Commons Attribution License (https://creativecommons.org/licenses/by/4.0/), which permits unrestricted reuse, distribution, and reproduction in any medium, provided the original work is properly cited.
Human Reproduction, 2026, 00, 1–28
https://doi.org/10.1093/humrep/deag032
Original Article
